# Functional Materials for ICE and IVUS Piezoelectric Transducers: A Review

**DOI:** 10.3390/s26072143

**Published:** 2026-03-31

**Authors:** Dayong He, Baihezi Ye

**Affiliations:** Ultrasonic Transducer Development Department, Shenzhen Mindray Bio-Medical Electronics Co., Ltd., Shenzhen 518057, China; yebaihezi@mindray.com

**Keywords:** ultrasound transducer, atherosclerosis, intracardiac echocardiography (ICE), intravascular ultrasound (IVUS), multifrequency ultrasound imaging

## Abstract

This review paper provides a comprehensive overview of the functional materials and assembly technologies used in intracardiac echocardiography (ICE) and intravascular ultrasound (IVUS) transducers. ICE and IVUS are advanced medical imaging technologies that play significant roles in the diagnosis and treatment of cardiovascular diseases, involving material selection and fabrication processes for miniature piezoelectric ultrasonic transducers. The review begins with an introduction to the principles and applications of ICE and IVUS, highlighting their advantages over other imaging modalities, then delves into the materials and assembly processes of the transducers, presenting the mainstream trends and research progress in various directions in this field in recent years. Finally, the paper summarizes the future technological development and clinical application trends of ICE/IVUS ultrasonic transducers.

## 1. Introduction

Both intracardiac echocardiography (ICE) and intravascular ultrasound (IVUS) are advanced medical imaging technologies that play significant roles in the diagnosis and treatment of heart and blood vessel diseases.

ICE involves placing an ultrasound transducer within the heart chamber through a catheter to emit and receive ultrasonic signals, providing real-time imaging of the heart’s anatomical structure and hemodynamics. It is widely used in various fields such as cardiac electrophysiology treatment, minimally invasive valve disease treatment, cardiac pacing treatment, and monitoring during congenital heart disease occlusion procedures. Compared to medical imaging technologies such as digital radiography (DR), computed tomography (CT) and magnetic resonance imaging (MRI), the advantages of ICE include no radiation, low risk, simple operation, and effective reduction in X-ray usage, which reduces radiation for both patients and medical staff. Even compared to transthoracic echocardiography (TTE) and transesophageal echocardiography (TEE), which are also ultrasound medical imaging technologies, ICE has its unique advantages as follows [1]: in guiding interventional procedures, ICE provides high-resolution images of cardiac structures by imaging from within the heart [2,3,4]. ICE does not require general anesthesia, reducing the involvement of echocardiographers and anesthesiologists [5]; ICE can be operated by an interventional cardiologist performing the procedure, which helps maintain continuity of information during surgery and enhances procedural efficiency [5]; and it effectively avoids the esophageal or gastrointestinal tract injuries that may be caused by TEE [6].

IVUS is a powerful imaging technology that has revolutionized the diagnosis and treatment of cardiovascular diseases. Similarly to ICE probes, IVUS involves the use of catheter-mounted ultrasound transducer(s) to provide detailed images of the interior of blood vessels, particularly coronary arteries. This technology has significantly enhanced the understanding of atherosclerosis and has become an essential tool in interventional cardiology. IVUS is widely used to assess the severity of coronary artery disease. It provides real-time high-resolution images of the vessel wall and lumen, which is crucial for determining the appropriate treatment strategy. IVUS is especially regarded as the gold standard for intracoronary imaging guidance in percutaneous coronary intervention (PCI) [7], including stent placement [8], balloon angioplasty [9], and other interventional procedures.

In percutaneous coronary intervention (PCI) for coronary calcific lesions, IVUS plays a crucial role. The ULTIMATE III trial demonstrated that the 1-year target lesion revascularization rate (TLR) of IVUS-guided drug-coated balloon angioplasty was 5.2%, significantly lower than 9.7% in the angiography-guided group. Its advantages stem from three core mechanisms: firstly, IVUS identifies calcified plaques through the characteristic of high echo accompanied by shadow (calcification curvature > 180° requires pre-treatment); secondly, after the procedure, it is necessary to confirm good stent apposition (no gap between the stent strut and the vessel wall) and adequate dilation (minimum lumen area ≥ 6 mm^2^); finally, it offers real-time monitoring of complications (such as linear anechoic area at the stent edge indicating endocardial tear requires immediate balloon re-expansion) [10].

Several comprehensive reviews have been published on IVUS imaging in recent years. Peng et al. [11] provide an extensive examination of ultrasound transducers for IVUS applications, focusing on piezoelectric transducers, piezoelectric micromachined ultrasound transducers (PMUTs), and capacitive micromachined ultrasound transducers (CMUTs), with particular emphasis on recent advances in piezoelectric materials and configurations. Sung et al. [12] present a detailed review of mechanically rotating IVUS transducers, systematically discussing the history, diagnostic indicators, and technical challenges in developing single-frequency and multifrequency mechanical IVUS transducers. Additionally, Leuzzi et al. [13] recently summarized the clinical applications of ICE in structural heart interventions, with emphasis on catheter design and procedural guidance. However, these existing reviews primarily focus on either IVUS or ICE as separate technologies, with limited attention to the convergence of functional materials and technologies that underpin both modalities. Specifically, a systematic comparison of piezoelectric materials, matching layers, backing materials, and fabrication processes across both ICE and IVUS transducers remains lacking. Furthermore, the miniaturization challenges, dual-frequency integration strategies, and the transition from 2D to 4D (real-time 3D) imaging—critical trends shaping the next generation of both technologies—have not been comprehensively addressed in a unified framework.

This review addresses these gaps by providing a comparative analysis of functional materials in ICE and IVUS transducers, highlighting their shared fundamental principles and distinct technical requirements. Unlike previous reviews that treat these technologies in isolation, we systematically summarize the selection and optimization strategies of piezoelectric materials, matching layers, backing layers, and catheter sheath materials for both applications, with attention to the new demands on material performance posed by emerging trends such as 4D imaging and dual-frequency integration. By synthesizing material advances across both fields, this review aims to provide guidance for the development of next-generation miniature ultrasound transducers.

## 2. ICE and IVUS Transducers

### 2.1. ICE/IVUS Piezoelectric Transducers: Typical Structure and Development Trends

**Figure 1:**
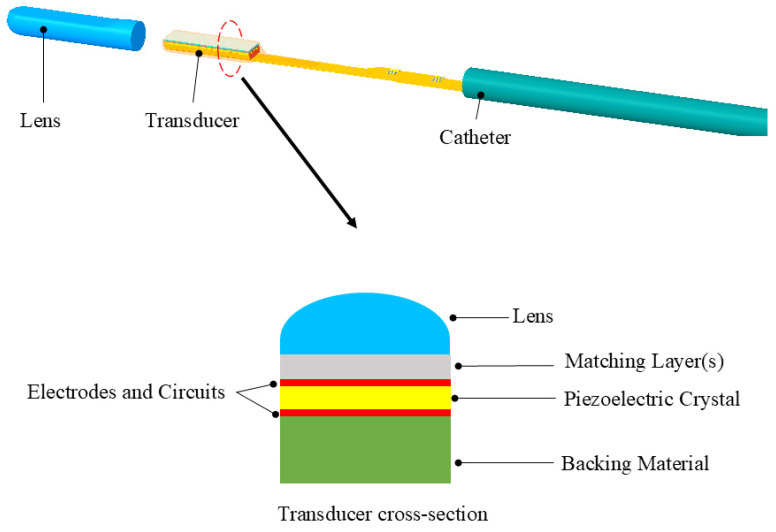
A typical composition of an ICE transducer, including cross-section scheme. Note: the cross-section reveals critical components: piezoelectric element generating ultrasound; acoustic lens for beam focusing; matching layers reducing impedance mismatch; backing layer damping residual vibrations.

Limited by the volume constraints of intracardiac/intravascular catheters, ICE and IVUS piezoelectric transducers represent typical miniaturized medical ultrasound devices. Taking the ICE transducer as an example, as shown in Figure 1, a typical medical ultrasonic transducer usually consists of main components such as piezoelectric crystals, acoustic lens, matching groups, electrodes and circuits, backing layers, etc. Due to limitations in volume or manufacturing processes, the structural composition in ICE and IVUS may be simplified to some extent.

**Figure 2:**
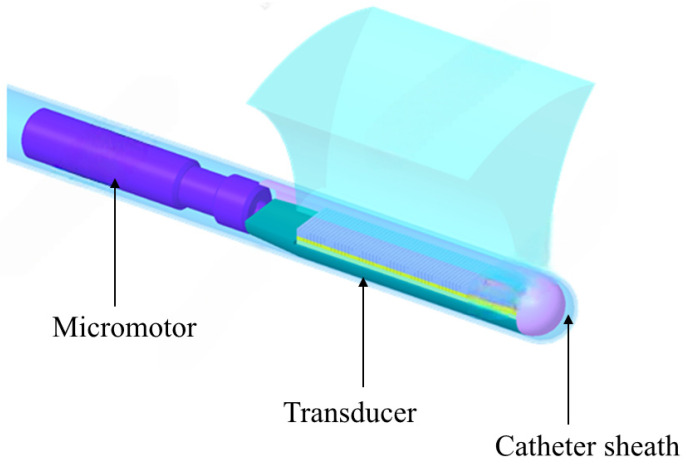
A simple schematic diagram of a micro-motor-driven 4D ICE.

Currently, 2D ICE is well-established, while 4D (real-time 3D) ICE represents the future direction with a broader imaging field of view and higher imaging quality, providing real-time three-dimensional reconstruction that aids in enhancing the precision and safety of surgical procedures. Compared with 2D ICE, 4D ICE can provide real-time three-dimensional volumetric images through micro-motors [14] or beamforming technology [15]; it allows for the observation of cardiac structures from multiple angles and supports multiplanar and imaging rapid switching between imaging planes [16]—this enhances surgical efficiency and reduces surgical risks. As presented in Figure 1 and Figure 2, 4D ICE achieves its functionality through two technical approaches: the rotation scanning driven by micro-motors provides mechanical three-dimensional reconstruction; and the electronic phased-array beamforming technology enables electronic scanning, thereby avoiding the reliability risks associated with moving parts. Moreover, 4D ICE catheters are suitable for more complex structural heart disease interventional surgeries, such as left atrial appendage (LAA) [17], mitral valve repair [18,19] and transcatheter aortic valve replacement (TAVR) [20,21].

**Figure 3:**
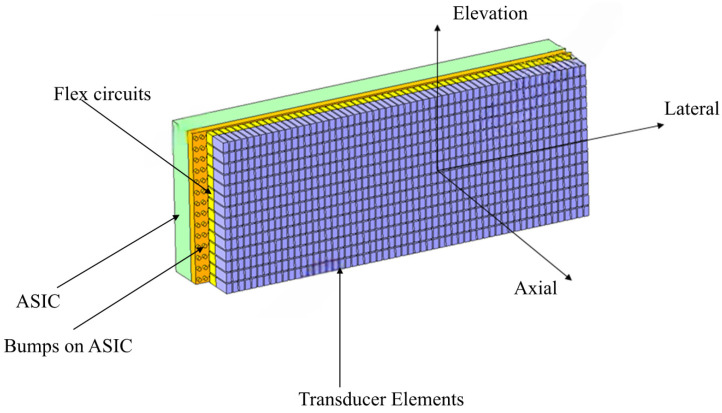
A simple schematic diagram of an electronic phased-array scanning 4D ICE transducers.

A multicenter study involving 200 atrial fibrillation patients demonstrated that 4D ICE-guided left atrial appendage (LAA) occlusion achieved a 98.5% success rate, significantly higher than the 92.3% success rate of TEE-guided procedures [22]. The core mechanism involves three-dimensional reconstruction during preoperative evaluation using 4D ICE, as shown in Figure 2 and Figure 3. This allows for precise measurement of the LAA orifice diameter with ≤1 mm error and depth—a circular hypoechoic area at the image center surrounded by myocardial tissue echogenicity. For a 22 mm orifice diameter, a 24 mm occluder is selected with 10% expansion space reserved. During intraoperative guidance, 4D ICE displays real-time occluder waist alignment with the LAA orifice. Good adhesion shows no echo gap; gaps > 1 mm require retraction. Postoperative verification through multi-plane switching (0°–90°) confirms occluder stability without displacement, contrast leakage, or strong contrast agent echogenicity into the LAA. In this study, the 4D ICE group had only 2.1% residual shunt < 3 mm postoperatively, significantly lower than the TEE group’s 7.8%. The procedure was shortened by 23 min due to a reduced need for general anesthesia preparation and better coordination with TEE teams [22].

Similar to ICE transducers, IVUS transducers can be classified into the mechanical rotary type [23] (which usually uses a single high-frequency crystal and easily achieves high-resolution imaging) and the electronic phased-array scanning type [11] (without micro-motor design, with better image stability). The former is suitable for small catheters due to its rotating sealing structure [12]; the latter can avoid non-uniform rotational distortion (NURD) and off-axis errors by its stationary design [24], and provides a wider depth of field.

With the advancement of IVUS technology, the miniaturization of IVUS transducers, the assembly of dual/multi-frequency IVUS [25,26], and the integration of IVUS with other imaging technologies such as optical coherence tomography (OCT) [27] have gradually become important trends in the development of IVUS. Dual-frequency transducer superharmonic IVUS technology has undergone rapid development from basic design to system integration since Ma et al. first proposed a miniaturized prototype (6.5 MHz/30 MHz) in 2014 [28]. Early research established that stacked configurations outperform interleaved layouts and significantly improve the contrast-to-tissue ratio (CTR) and axial resolution by reducing transmission frequency to 5 MHz and employing PMN-PT composite materials [29]. The key breakthrough in 2016–2017 was the introduction of lateral mode transmitters, enabling catheter sizes below 3 Fr (French, 1 Fr ≈ 0.33 mm diameter, a standard unit for catheter sizing) and the development of the first 360-degree dual-frequency superharmonic IVUS cylindrical array capable of real-time imaging [30]. In 2013, Maresca et al. completed the construction of an integrated imaging system with a 1–60 MHz receiving bandwidth and a 30 dB dynamic range in the superharmonic mode [31]. This technology exploits the nonlinear acoustic response of microbubbles excited at low frequencies while detecting higher-order harmonics (4th–15th) at high frequencies, achieving clear imaging of 200 μm-level microvessels in phantom and ex vivo experiments. However, challenges remain in noise suppression, in vivo clinical translation, and further miniaturization. The field has entered a relatively quiet period since 2018, with latest developments mostly reported in conference proceedings or commercial translation stages. The evolution of dual-frequency IVUS from 2014 to 2018 follows a predictable innovation S-curve: initial proof-of-concept (6.5 MHz/30 MHz [28]), followed by performance optimization [29] via stacked configurations and PMN-PT composites (5 MHz/30 MHz, improved contrast-to-tissue ratio), then a miniaturization breakthrough (lateral mode transmitters, catheter size less than 3 Fr) [30], and finally system integration (1–60 MHz receiving bandwidth, 30 dB dynamic range) [32]. The plateau post-2018 suggests technical maturity or market consolidation, with current efforts focused on clinical translation rather than fundamental material innovation. This pattern mirrors the historical trajectory of ICE from 2D to 4D imaging, from mechanical rotation [14] to the electronic phased array [15], indicating that both modalities face similar late-stage engineering challenges (thermal management, ASIC integration, cost reduction) rather than early-stage material discovery. The convergence is further evidenced by a shared reliance on PIN-PMN-PT single crystals, 1–3 composites, and MEMS-compatible fabrication across both ICE and IVUS development pipelines.

The evolution of ICE and IVUS transducers from simple 2D imaging to sophisticated 4D real-time volumetric reconstruction and dual-frequency imaging represents a significant trend toward functional integration and miniaturization. This complexity imposes increasingly stringent demands on all constituent materials. Piezoelectric crystals must exhibit broader bandwidth and higher electromechanical coupling coefficients to enable multi-frequency operation and harmonic imaging, driving the transition from conventional PZT to advanced single-crystal PMN-PT or composite materials. Matching layers require precisely graded acoustic impedance with minimal attenuation to accommodate wider frequency ranges, necessitating development of nano-composite materials with tunable properties. Backing layers face the challenge of providing adequate damping without excessive bulk in catheter-constrained geometries, prompting innovations in epoxy-based composites with optimized filler architectures. The acoustic lens must achieve precise beam focusing across multiple frequencies while maintaining flexibility for catheter navigation, requiring advanced molding techniques and materials with stable acoustic properties under dynamic bending. Furthermore, the integration of micro-motors for mechanical 4D imaging or phased-array electronics for electronic beam steering introduces additional material challenges in biocompatible encapsulation, thermal management, and reliable interconnection within sub-millimeter catheter diameters. As dual-modality devices (IVUS-OCT) and superharmonic imaging systems continue to emerge, the convergence of acoustic, optical, and mechanical functionalities will undoubtedly necessitate breakthroughs in multifunctional materials, heterogeneous integration processes, and nanoscale fabrication technologies to meet the rigorous performance and safety standards of next-generation interventional imaging.

### 2.2. The Piezoelectric Materials Applied in ICE and IVUS Transducers

As the core of the acoustic–electric signal conversion, piezoelectric materials critically determine three key performance metrics of ICE/IVUS transducers: (1) center frequency, governing axial resolution; (2) fractional bandwidth, determining pulse length and tissue contrast; and (3) electromechanical coupling efficiency, affecting sensitivity and penetration depth. The ideal performance targets differ between ICE and IVUS: ICE requires moderate frequencies (5–10 MHz) with a wide bandwidth for deep cardiac imaging, while IVUS demands high frequencies (20–100 MHz) with superior resolution for microvascular structures. This section discusses how different material systems address these performance goals.

#### 2.2.1. Achieving Moderate Frequencies with Reliable Performance: PZT Ceramics in ICE Applications

For ICE transducers operating at 5–10 MHz, PZT ceramics provide a mature solution balancing performance and manufacturability. Lee et al. [14] fabricated a 10 Fr micro-motor-driven 4D ICE catheter using commercial PZT, achieving a −6 dB bandwidth of 61% with beam widths of 1.8° (azimuth) and 6.6° (elevation). The same team later assembled an electronic phased-array scanning 4D ICE catheter with another commercial PZT variant, reaching 60% bandwidth [15]. These results demonstrate that PZT ceramics adequately meet ICE requirements for bandwidth (~60%) and beam control, though their relatively lower electromechanical coupling coefficient (*kt* ≈ 0.5) limits further bandwidth extension.

#### 2.2.2. Pursuing High-Frequency Operation and Wide Bandwidth: PMN-PT Single Crystals for IVUS

For high-frequency IVUS (>40 MHz), where axial resolution targets <50 μm, PMN-PT single crystals have become the material of choice due to their superior electromechanical coupling (*kt* > 0.6) compared to PZT. Shung and co-workers synthesized free-standing PMN-PT single-crystal films, fabricating an 82 MHz IVUS transducer with 65% bandwidth and 35 μm axial resolution [33]. The same team achieved a 72% bandwidth at 45 MHz using a bulk single-crystal design [34]. Ma and Cao [35] reported a 45 MHz PMN-PT single-element transducer with 61% bandwidth and 40 µm axial resolution. These studies establish that PMN-PT single crystals enable higher operating frequencies with maintained or improved bandwidth compared to PZT, directly addressing IVUS requirements for microstructure visualization.

#### 2.2.3. Maximizing Bandwidth Through Composite Architectures

To overcome the bandwidth limitations of monolithic materials, 1–3 piezoelectric composites have been developed, offering higher *kt* and lower acoustic impedance (4–25 MRayls vs. >30 MRayls for bulk ceramics), reducing the acoustic mismatch between the transducer and human tissue (about 1.5 MRayls) [36,37,38]. Han et al. [39] fabricated a phased-array ICE transducer using a 1–3 composite with 55% bandwidth, optimizing uniformity through beveling. For IVUS applications, Li et al. [40] developed a PIN-PMN-PT single-crystal 1–3 composite achieving 86% bandwidth at 41 MHz—the widest reported bandwidth for IVUS—demonstrating that composite architectures can simultaneously address high-frequency operation and ultra-wide bandwidth requirements, while Yuan et al. [41] utilize deep reactive ion etching to achieve kerf widths of ~4 µm, yielding 77% bandwidth at 41 MHz and 86% bandwidth at 60 MHz. These advances highlight the importance of microscale structuring in optimizing the trade-off between frequency and bandwidth.

#### 2.2.4. Lead-Free Alternatives: Balancing Environmental Requirements with Performance Gaps

In IVUS application, lead-based piezoelectric materials, such as PZT ceramics, PMN-PT, and PIN-PMN-PT single crystals, are widely used in IVUS transducers due to their excellent piezoelectric properties and mature manufacturing techniques. However, environmental concerns associated with lead-containing materials and the potential health hazards during manufacturing and disposal processes have prompted researchers to seek lead-free alternatives. Yan et al. [42] fabricated a 30 MHz IVUS transducer using lead-free BZT-50BCT ceramic, which exhibits a high dielectric constant (*εr*/*ε*0) of approximately 2800 and a piezoelectric coefficient (*d_33_*) of around 600 pC/N. Liu et al. [43] report a non-Pb pseudo-binary ferroelectric system, (1-*x*)Ba(Zr_0.2_Ti_0.8_)O_3_-*x*(Ba_0.7_Ca_0.3_)TiO_3_, which shows piezoelectric properties comparable to PZT when x = 50; based on this material, a 30.5 MHz IVUS transducer was fabricated and exhibits a −6 dB bandwidth of 53% and an insertion loss of 18.7 dB. Zhu et al. [44] utilized lithium-doped KNN (KNLN) film to develop a 50 MHz IVUS transducer; the film possesses an electromechanical coupling coefficient (*kt*) of 0.44 and the transducer reached a −6 dB bandwidth of 61.5%. While these lead-free materials demonstrate promise, their bandwidth (53–61.5%) and electromechanical coupling remain below those of PMN-PT single crystals (65–86%), indicating continued performance gaps for high-frequency, wide-bandwidth applications. Overall, while lead-based materials still dominate in IVUS transducers, the research and development of lead-free materials are making progress and hold the potential to provide more environmentally friendly alternatives with reduced toxicity concerns during material processing and device manufacturing.

Appropriate piezoelectric materials are the starting point for the design of ultrasonic transducers. For small medical ultrasonic transducers such as ICE and IVUS, the selection of piezoelectric materials needs to be more carefully considered. Table 1 summarizes the performance comparison across material systems, illustrating the progression from PZT ceramics (adequate for ICE) to PMN-PT single crystals and composites (optimal for high-frequency IVUS), alongside the emerging but still developing lead-free alternatives.

#### 2.2.5. Comparative Framework for Material Selection

The diverse piezoelectric material systems reviewed above can be positioned within a three-dimensional trade space defined by the electromechanical coupling coefficient (*kt*), fractional bandwidth, and manufacturing readiness (Table 1). This framework reveals distinct occupancy patterns: PZT ceramics occupy the quadrant of moderate (*kt* ≈ 0.5) and bandwidth (55–65%) with high manufacturing maturity, aligning with ICE requirements for proven reliability and cost-effectiveness. PMN-PT single crystals and 1–3 composites extend toward high (> 0.60) and ultra-wide bandwidth (> 70%), satisfying IVUS demands for high-frequency resolution despite increased fabrication complexity. Lead-free alternatives currently populate an intermediate zone, indicating a developmental trajectory toward environmental compliance without catastrophic performance penalty. Notably, the convergence of ICE and IVUS evolution—both demanding enhanced bandwidth for harmonic imaging and miniaturized dimensions for catheter compatibility—drives shared material innovations, particularly in composite microfabrication and MEMS integration, that transcend the traditional boundary between these modalities.

#### 2.2.6. Fundamental Trade-Offs in Miniature Transducer Design

The axial resolution Δ*z* limit in a pulse-echo ultrasound is governed by the spatial extent of the acoustic pulse, which can be approximated by ([14]):

Δ*z* ≈ *c*/(2 · *BW*) = *c*/(2 · *f_c_* · *BW*_fractional_) (1)

where *c* ≈ 1540 m/s is the acoustic velocity in soft tissue, *f_c_* is the center frequency, and *BW*_fractional_ is the fractional bandwidth. For ICE operating at *f_c_* = 6 MHz with *BW* = 60%, this yields Δ*z* ≈ 214 μm; for IVUS at *f_c_* = 40 MHz with *BW* = 70%, Δ*z* ≈ 39 μm, consistent with the experimentally reported values in Table 1.

The penetration depth *δ* is constrained by frequency-dependent attenuation following [39]:

*δ* ≈ 1/2*α*(*f_c_*) ∝ 1 / (*f_c_*)^1.5^ (2)

where *α*(*f*) = *α_0_f*^n^ with *n* ≈ 1.0–1.5 for soft tissue. This inverse relationship dictates *δ* ≈ 10 cm for ICE versus *δ* ≈ 2–5 mm for IVUS, establishing their distinct clinical niches—deep cardiac imaging versus superficial vascular assessment—despite convergence in material requirements for wideband, miniature transduction [48].

### 2.3. Lens/Catheter Sheath Materials

ICE/IVUS transducers are typically housed within polymer catheter sheaths. To match the acoustic impedance of human tissue or blood (~1.5 MRayls) [49] and considering biocompatibility, polymers with lower acoustic impedance—such as polyether block amide (PEBAX), polyurethane (PU), and polymethylpentene (TPX)—are commonly selected as the material for the catheter sheath; the acoustic properties at around body temperature of these polymers are presented in Table 2.

1. Temperature Dependence: Polyurethane materials exhibit a negative temperature coefficient of sound velocity (unlike water, blood, and muscle, which show positive coefficients). For RP 6400, Bacon 430, and Santoprene, the velocity decreases by approximately 60–124 m/s when temperature increases from 25 °C to 37 °C.
2. Measurement Methods: Data from Guess and Campbell [49] were obtained using a Fourier transform pulse-echo method with temperature controlled at 37 °C ± 0.05 °C. Data from Stephens et al. [51] were derived from array transmission measurements; specific methodology was not detailed, and direct quantitative comparison with pulse-echo data should be made with caution.
3. Acoustic Impedance Calculation: Z = ρ × V_l_, calculated from measured density and sound velocity values. Minor discrepancies with original publications may reflect rounding or measurement uncertainties.
4. Hardness Designation: Shore hardness values: D = Shore D scale (harder materials), A = Shore A scale (softer materials).

For the micro-motor-driven 4D ICE/IVUS transducers, it is necessary to fill the sheath with an acoustic coupling medium to ensure the rotation of the transducer and the transmission of ultrasound; common acoustic coupling media include propylene glycol glycerol, saline solution, water, silicone oil, and specialized coupling gels [53]. To prolong the life of the transducer and minimize bubble interference, it is advisable to inject coupling fluid into the catheter sheath before using ICE/IVUS catheters and to thoroughly de-bubble it [14]; this process ensures that the coupling fluid, which serves as a medium for transmitting ultrasound waves, is free of air bubbles that could otherwise interfere with the imaging process, and avoids unnecessary corrosion of the transducer by the coupling medium. The higher the center frequency of the transducer possesses, the more severe the acoustic attenuation of the catheter sheath will be [12,25], and this will have a greater impact on image quality; this negative impact is more pronounced in IVUS catheters with micro-motor-driven transducers. To mitigate the performance loss caused by this phenomenon, in addition to using materials with lower acoustic attenuation to make the catheter sheath rotate as much as possible, the thickness of the sheath should also be minimized [54]; however, this may pose a challenge to the reliability of the IVUS catheter. In addition, the IVUS transducer can also be tilted at a small angle [55] (0–8°) to avoid the direct reflection of ultrasound waves from the catheter sheath to the transducer. This helps to reduce the interference of reflected signals with imaging.

In electronic phased-array intracardiac echocardiography (ICE) transducers, operating frequencies typically range from 5 to 10 MHz (commercial systems: Siemens AcuNav 8–10 MHz, Boston Scientific ViewFlex 5.5–10 MHz, St. Jude MediGuide 7.5 MHz). At these frequencies, acoustic attenuation of catheter sheath materials remains low (<2 dB/cm/MHz for Pebax at 37 °C) [49], allowing the sheath to serve a dual function as protective encapsulation and acoustic lens. Stephens et al. [51] demonstrate that optimal beam performance requires lens sound speed approximately 100–200 m/s below tissue velocity (~1480 m/s vs. ~1590 m/s for cardiac muscle at 37 °C). Materials with near-tissue-matched velocities enable full-round lens profiles (~1.2 mm radius at 7.25 MHz), while higher-velocity materials require tapered designs with compromised surface smoothness. In similar applications, it is necessary to treat the adhesive and the surface of the transducer to avoid bubbles, voids, and deformation of the lens [15].

For intravascular ultrasound (IVUS) transducers, clinical frequencies range from 20 to 60 MHz (commercial systems: Philips Volcano Eagle Eye 20 MHz, Boston Scientific iLab 40–60 MHz) [11], with research prototypes exceeding 100 MHz [56]. The 5–10× frequency increase fundamentally alters precision requirements. Cannata et al. [57] demonstrate at 35 MHz that kerf width must scale down to ~14 μm (~50 μm at 7 MHz) using mechanical dicing; element pitch reduces to 50 μm (1.2λ), and matching layer thickness drops to ~19 μm. Cable effects become critical: at 35 MHz, cables introduce ~0.61 dB/m attenuation, requiring impedance matching to transform high element impedance to 50 Ω electronics.

Higher-frequency IVUS will face more severe fabrication precision challenges. As frequencies increase to 40–60 MHz and above, wavelengths become progressively shorter, requiring correspondingly smaller kerf widths and element pitches that approach the limits of conventional mechanical processing. Matching layer thickness must decrease to <10 μm, demanding tighter thickness control. Cable losses increase sharply with frequency, severely limiting signal-to-noise ratio and imaging depth. At 100 MHz-class frequencies, existing bulk piezoelectric ceramics and processing techniques will encounter fundamental limitations, necessitating alternative technical approaches. The frequency-squared increase in attenuation precludes thick lenses for IVUS. Consequently, geometrical focusing via spherically curved transducer surfaces is employed [58], and the outcome is a geometric requiring finer sphericity tolerance in mandrel fabrication and sub-micron matching layer precision [48]. Such structures will pose challenges to the catheter’s hermeticity and biocompatibility. Therefore, in order to meet the relevant medical device industry standards [59,60], the selection of transducer-functional materials and the choice of the encapsulation process need to be carefully considered.

### 2.4. The Matching Layers

When sound waves propagate vertically between two media with different impedances, the reflection coefficient *R*, which represents the ratio of the amplitude of the reflected wave to the amplitude of the incident wave, is given by the formula below [61]:

(3) $$R=|\frac{Z_{2}-Z_{1}}{Z_{2}+Z_{1}}|,$$

where *Z*_1_ and *Z*_2_ are the acoustic impedances of the two media. Correspondingly, the formula for calculating the transmission coefficient *T* is [61]:

(4) $$T=|\frac{{2Z}_{2}}{Z_{2}+Z_{1}}|,$$

The energy reflection coefficient *R_E_* and the energy transmission coefficient *T_E_* represent the ratio of the energy of the reflected wave to the energy of the incident wave, and the ratio of the energy of the transmitted wave to the energy of the incident wave, respectively. Their calculation formulas are [61]:

(5) $$R_{E}=R^{2}={\left(\frac{Z_{2}-Z_{1}}{Z_{2}+Z_{1}}\right)}^{2},$$

(6) $$T_{E}=\left({1-R}^{2}\right)=\frac{{4Z_{1}Z}_{2}}{{\left(Z_{2}+Z_{1}\right)}^{2}}$$

Ideally, the acoustic impedance of the piezoelectric layer should closely match that of the tissue to allow for the efficient transmission of ultrasound waves into the tissue. However, most piezoelectric materials, such as the commonly used ceramics, have a high acoustic impedance (>30 MRayls), which is significantly different from the acoustic impedance of human tissue (≈1.5 MRayls) If *Z*_2_ and *Z*_1_ differ significantly, the reflection coefficient will be large. The acoustic impedance of the matching layers lies between that of the piezoelectric element and the medium, which reduces the difference between *Z*_1_ and *Z*_2_, thereby decreasing the reflection coefficient. They serve as transitional media, reducing the reflected energy and increasing the transmitted energy as the sound waves propagate from the piezoelectric element to the media.

For the multilayer matching of transducers, many studies have already established corresponding models. The design of multilayer matching structures employs established acoustic transmission theories, primarily the Krimholtz–Leedom–Mattaei (KLM) equivalent circuit model and transmission line (TL) theory. The KLM model represents the piezoelectric element as a three-port network with acoustic ports at each face and an electrical port, enabling calculation of input electrical impedance and acoustic output under arbitrary loading conditions. For multilayer matching, each layer is modeled as a transmission line segment with characteristic impedance *Z_n_ = ρ_n_c_n_* and electrical length *θn* = 2 *πfd_n_*/*c_n_*, where *d_n_* is layer thickness [62,63,64,65]. For practical implementation, the quarter-wavelength matching condition (*d* = *λ/4*) at center frequency *f_c_* remains dominant, with layer impedances following the geometric mean approximation for double-layer structures: Z_1_ = (Z_piezo_ × Z_2_)^1/2^ and Z_2_ = (Z_1_ × Z_tissue_)^1/2^ [66]. These models enable predictive design prior to fabrication, reducing empirical iteration. 

For medical piezoelectric ultrasonic transducers with multilayer matching, it is common to apply multiple layers of materials with decreasing characteristic impedance on the surface of the piezoelectric material as matching layers [67]; for each layer of material, its thickness is usually one-quarter of the wavelength of the transducer in that material to achieve efficient transmission of acoustic energy [68].

Due to the lower center frequency and larger size, the choice of matching materials and the processing of ICE transducers are relatively simple. For matching layers with impedance over 10 MRayl, glass and ceramic-based bulk materials are commonly selected as matching layers. These materials typically have a high sound velocity (>4000 m/s), and their density, which is controlled by the composition of the material itself, usually ranges from 2 g/cm^3^ to 4 g/cm^3^ [69]. For matching layers with impedance in the range of 4–9 MRayl, researchers generally need to prepare composite materials on their own to meet the impedance requirements; for example, Zhou et al. [70] fabricated alumina/polymer nanocomposite films by a spin-coating process with alumina volume fractions ranging from 14 to 32%; the particle size of alumina is in the range of 10 to 40 nm, the thickness of the matching layer can be controlled by the rotation speed and the concentration of the solution, the acoustic impedance of these nanocomposite matching layers ranges from 2.8 to 5.1 MRayls. In Lee et al.’s works [14,15], their team frequently uses a metal-filled graphite material as the matching layer, which has a characteristic impedance of about 6.6 MRayl and can be used for the transmission of electrical signals. For matching layers with an acoustic impedance of less than 4 MRayl, common commercial plastics, such as ABS [15] and epoxy resin [71], can meet the requirements.

A 10 Fr catheter diameter would limit its application in small cardiac chambers. When occupying the chamber space, it may compress the ventricular wall and cause artifact-induced stenosis. Imaging may show thickened ventricular walls, which are actually caused by catheter compression. This limited visualization has multifaceted clinical impacts: in pediatric congenital heart disease interventions, it often leads to misjudgment of valve dimensions—such as overestimating pulmonary valve annulus measurements. Patients with narrow cardiac chambers might miss critical structures like mitral valve leaflets due to catheter obstruction. To address these issues, we propose developing a 7 Fr ultra-fine 4D ICE catheter using PIN-PMN-PT single-crystal materials and optimizing beamforming algorithms (as shown in Figure 2) to reduce self-obstructing areas of the catheter.

Due to the small size and high frequency of IVUS transducers, stringent requirements for acoustic attenuation and thickness precision limit the range of suitable matching materials. Silver-based conductive epoxy combined with Parylene represents one viable approach—as demonstrated by Lee et al. [72] with a 7.33 MRayl Ag-epoxy first layer and 2.59 MRayl Parylene second layer. The silver-epoxy mixture can be easily casted on the piezoelectric materials and grinded into a proper thickness; parylene coatings are prepared through a vacuum chemical vapor deposition (CVD) process, and the thickness can be precisely controlled by regulating the deposition time. Alternative strategies have been widely adopted depending on frequency and fabrication constraints. For instance, single-layer matching using E-solder (5.9 MRayl) has achieved 62% bandwidth at 43.5 MHz [73], and pure Parylene coatings serve as standalone matching layers in high-frequency (>100 MHz) designs where ultra-thin piezoelectric layers preclude composite matching [26]. Additionally, mass-spring acoustic matching segments [74] using sputtered metal and vapor-deposited polymer layers offer precise impedance tuning without powder-loaded epoxy. These variations underscore that matching layer design in IVUS is dictated by specific frequency, aperture, and manufacturing considerations rather than universal material standards. 

The application of matching layers can significantly enhance the acoustic performance of transducers and further optimize image quality. Generally, transducers with multilayer matching have advantages over those with single-layer matching. However, in practical design, it is necessary to consider the processing difficulty and corresponding costs and seek a balance between performance and the fabricating process. Table 3 summarizes the matching layer strategies employed in ICE and IVUS transducers.

### 2.5. The Backing Layer Materials

In ultrasonic transducers, the primary role of the backing layer is to absorb signals emitted from the back surface of the piezoelectric materials, preventing these signals from reflecting back into the transducer and causing interference, while also reducing the vibration time of the piezoelectric material to shorten the pulse width [76]. To achieve this, high-attenuation materials are typically installed on the back of the piezoelectric layer to effectively absorb the energy. It should be noted that, while high attenuation suppresses multiple reflections within the backing material, the primary reflection at the piezo/backing interface is governed by impedance mismatch and cannot be eliminated by attenuation alone; otherwise, stray signals may be introduced if the backing layer is too thin or impedance matching is inadequate.

To minimize signal ringing, the acoustic impedance of the backing layer can be matched with that of the piezoelectric material, but this will result in a significant amount of energy being transferred to the backing layer and lost, thereby reducing the sensitivity of the transducer. Therefore, a trade-off between sensitivity and bandwidth is necessary in the design. Generally, the acoustic impedance of the backing layer is slightly lower than that of the piezoelectric layer, which can increase sensitivity at the cost of slightly increased pulse length or signal ringing. This design optimization is very important in practical applications, especially in the fields of ICE and IVUS transducers, where the characteristics of the backing layer need to be adjusted according to specific requirements to meet different imaging needs and the space of the catheters are limited.

The mechanisms of acoustic attenuation in backing materials mainly involve several aspects [77]: First, ultrasonic waves lose energy due to scattering as they propagate through the medium, especially when encountering particles or inhomogeneities that scatter the waves in multiple directions, resulting in energy dispersion. Second, absorption losses of ultrasonic waves in the medium are also an important cause of attenuation, which are mainly caused by thermoelastic effects, viscous losses, inelastic hysteresis, and relaxation processes. Thermoelastic effects refer to the conversion of part of the ultrasonic wave energy into heat due to thermal expansion and contraction caused by the waves; viscous losses are due to the viscosity of the medium, which absorbs and converts the ultrasonic waves into heat during propagation; inelastic hysteresis and relaxation processes also lead to the absorption and transformation of ultrasonic wave energy. In addition, the properties of the polymer matrix, such as cross-linking and crystallinity, also have a significant impact on acoustic attenuation. Polymer matrices with low cross-linking usually have higher attenuation performance because they are more prone to thermal expansion and contraction, thereby increasing thermoelastic effects. The size and volume fraction of particles, as well as the interfacial properties between particles and the polymer matrix, also affect attenuation. Larger particles and lower volume fractions usually result in higher attenuation because they provide more scattering centers. Good adhesion between particles and the polymer matrix can reduce the scattering and reflection of ultrasonic waves, thereby reducing attenuation [78]. Overall, attenuation is the result of the combined action of multiple mechanisms, including scattering, thermoelastic effects, viscous losses, inelastic hysteresis, relaxation processes, and the characteristics of the polymer matrix and particles.

In typical ICE catheters, the total thickness of the transducer is usually only a few millimeters. After accounting for the matching layer, piezoelectric material, and necessary electrical connections, there is very limited space left for the backing material, so the attenuation coefficient should be high enough. In an article mentioned above, Stephens [51] and co-workers employed a high-attenuation backing material with an attenuation coefficient of 129 dB/cm at 10 MHz; however, the authors did not provide the synthesis method for this high-attenuation backing material. In Lee’s [14,15] works, the authors’ attempt to use a high-attenuation backing based on glass bubbles failed because it was mechanically weak, but they reflected most of the acoustic energy toward the front surface by introducing a high-acoustic-impedance de-matching layer (tungsten carbide, *Z* = 104 MRayl) on the back of the acoustic stack; as a result, they were able to complete the transducer design using a less-than-ideal backing material.

**Figure 4:**
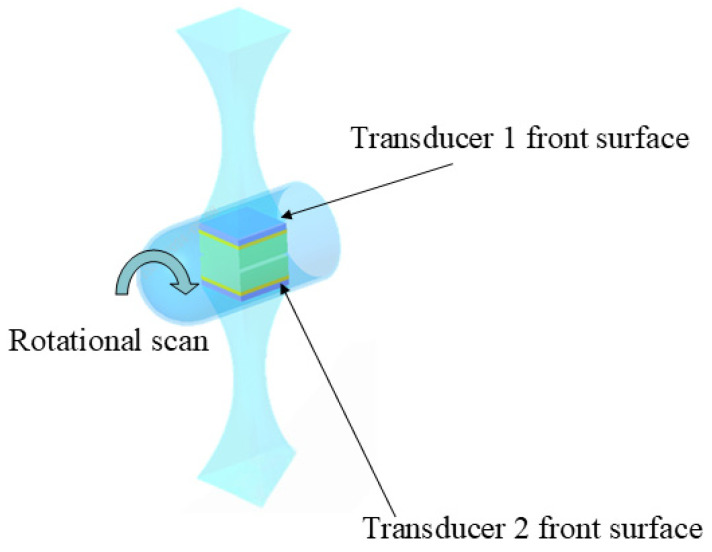
Illustration of back-to-back dual frequency IVUS transducer.

For IVUS transducers, due to their small size, the introduction of a high-attenuation backing can reduce the thickness of the acoustic stack; however, non-conductive backings typically pose difficulties in the design of complex-structured transducers. Therefore, a careful trade-off between the acoustic performance and electrical conductivity of the backing needs to be considered. Kim et al. [79] introduced a novel high-attenuation backing layer for miniaturized ultrasound imaging transducers; the material composes glass bubbles, polyamide resin, and tungsten powder and achieves an attenuation coefficient of over 160 dB/cm at 5 MHz, five times higher than that of the commonly used silver-based conductive epoxy for high-frequency ultrasound transducers. Additionally, the material possesses suitable acoustic impedance (4.6 MRayl) and sufficient hardness to meet the requirements of miniaturized transducers. Experimental results demonstrate that an intravascular ultrasound (IVUS) transducer using this backing layer reduces the amplitude of the signal returned from the backing layer by 1.8 times—this means the signal amplitude with a high-attenuation backing layer was reduced to approximately 56% of the amplitude with the conventional backing and attenuates ringing by 6 dB. In terms of axial resolution, the transducer with the novel backing layer shows a 30% improvement compared to those with silver-based conductive epoxy backing. In intravascular ultrasound (IVUS) transducers, the use of conductive epoxy as the backing layer is a mainstream practice [26,46,58,80,81,82,83,84,85,86] because it is compatible with a flexible electrical connection design. For example, Sung et al. [46] designed a novel IVUS transducer with asymmetric electrodes by using a combination of conductive and non-conductive backing layers. This design minimizes the impact of adhesive on transducer performance; the electrical impedance of the new transducer is 23 Ω at 60 MHz, with a center frequency of 60.2 MHz and a −6 dB bandwidth of 57.2%; the received signal amplitude is 0.62 Vpp, which is sufficient for detecting lesions within blood vessels; compared to conventional transducers, the new transducer shows a 15%, 12%, and 10% increase in maximum penetration depth at −3 dB, −6 dB, and −10 dB pressure levels, respectively; and the pressure distribution of the new transducer is more uniform, resulting in a more consistent signal-to-noise ratio (SNR) and significantly reduced beam distortion. Ma et al. [26] fabricated three prototypes of multi-frequency IVUS catheters with two transducers (35/90 MHz, 35/120 MHz, and 35/150 MHz) with the conductive backing layers. The two transducer elements were arranged in back-to-back configuration for automatically facilitating image co-registration at the same longitudinal artery location (Figure 4); the insulation between the two transducer signals was achieved simply by adhering a polyimide layer between the conductive backing layers. This structure allows the two transducers to generate images of the same cross-section within a single frame and effectively utilizes the space within the catheter. It features a small outer diameter and a short rigid segment, which are advantageous for clinical applications.

For small medical ultrasound transducers such as ICE and IVUS, the limited thickness space necessitates the acoustic stack to be designed as thin as possible. The integration of dual-frequency IVUS capabilities and accompanying electrophysiological accessories within the catheter further restricts the space available for the transducer. This imposes more stringent demands on the performance of the backing layer. Simultaneously, the electrical connection design requires the backing material to be highly conductive. Therefore, the development of conductive, high-attenuation backing materials will be essential for enhancing ultrasound catheter performance and simplifying catheter construction; excellent backing performance can save substantial space for the miniaturization of high-performance transducers. Table 4 examines the interplay between catheter outer diameter (OD), array channel count, and cable strategy in ICE and IVUS transducers, revealing critical engineering trade-offs that determine device feasibility and imaging performance.

### 2.6. The Connection and Isolation in ICE and IVUS Transducer, Acoustically and Electrically

For the development and manufacturing of ultrasonic transducers, the efficient transmission of acoustic signals (e.g., from the piezoelectric crystal to the matching layers) and the isolation of these signals (e.g., the isolation of crosstalk between elements) are key to the realization of the transducer’s acoustic design. Correspondingly, the extraction and insulation of the transducer’s electrode signals determine whether the device can function properly. 

Due to the lower operating frequency and larger size constraints, compared with IVUS transducers, ICE transducers have more options for electrical signal connections. Typically, piezoelectric materials with conductive coatings are bonded to a Flexible Printed Circuit (FPC) to lead out one electrode, while the other electrode is achieved through a conductive matching layer, conductive coatings on the matching layer [15], or a thin metal foil [89]. The acoustic properties of the FPCs cannot be ignored in acoustic models, the thickness and proportion of the metallic and polymer phases of the FPC need to be strictly designed. In fact, in traditional 1D linear array ultrasonic transducers, some researchers have achieved the function of a matching layer by adjusting the ratio of copper to polyimide and the number of stacked layers in the FPC [75]. In electronic phased-array scanning 4D ICE transducers, to achieve subarray beamforming, it is necessary to control the electrical excitation of individual sub-elements on a two-dimensional matrix array; the diameter of the catheter limits the integration of many-channel cables in the catheter, but the use of application-specific integrated circuits (ASICs) in the transducers has solved this problem. With the advancement of high-voltage, mixed-signal ASIC technology [90], the circuit size of each element has gradually approached the size of the piezoelectric material elements, which has led to the emergence of 4D ICE transducers with similar design [88]. In addition to reducing the probe volume, the ASIC can also effectively amplify the received signals, preventing signal attenuation caused by cable loading [91,92]. The acoustic properties of the ASIC are equally important and cannot be ignored; in acoustic simulation, it is typically treated as silicon with an acoustic impedance of about 19.7 MRayls. [15]

The array division of ICE transducers involves separating the matching layer, piezoelectric material, and corresponding electrodes into individual elements. Since this process involves cutting through multiple layers and different materials, dicing saws are typically used [39], which are devices used to cut processed materials into small pieces (such as chips or grains) and are widely used in the fields of semiconductors, microelectronics, and optoelectronics. They achieve cutting with high-speed rotating spindles equipped with extremely thin diamond blades or diamond wires. In the field of ultrasonic transducers, it usually only involves straight-line cutting; however, to ensure cutting accuracy and process feasibility, it is required that each layer of the acoustic stack has good planarity and that the layers are firmly bonded together. This is especially true for ultrasonic transducers that use adhesive bonding processes. There is a certain contradiction between ensuring the firmness of the adhesive layer and minimizing the presence of the adhesive layer in the acoustic model, which needs to be carefully balanced. The acoustic and electrical connection designs of the transducer collectively determine the position and method of saw cutting. In some studies, to ensure complete electrode lead-out, it may be necessary to perform bonding on certain components of the transducer after cutting, followed by a second cutting of the corresponding elements [15]. The gaps between the elements after dicing are referred to as the kerf. Theoretically, an air kerf (a kerf with no filling material) can ensure the acoustic independence of the elements and avoid crosstalk of acoustic and electrical signals. However, in practice, to ensure the mechanical strength of the transducer, epoxy resin is often used to fill the kerf. To further reduce crosstalk, some researchers even use insulating backing materials to fill the kerf in an attempt to completely absorb the lateral vibrations between elements [51].

For IVUS transducers, the extremely small size and high operating frequency dictate that the electrical connections and transducer structure must be simplified as much as possible. The use of a conductive matching layer and a parylene insulation layer as the matching layer, as well as the method of leading out electrodes using a conductive backing layer, have been summarized above in Section 2.4 and Section 2.5. For insulating materials, if it is necessary to lead out the electrodes of IVUS transducers, this can be achieved through metal coatings, typically Cr/Au or Ti/Au [28]; however, for high-frequency transducers, the thickness of the metal layer needs to be more strictly controlled—usually, a metal layer of just a few tens of nanometers is sufficient to achieve a reliable electrical connection. This process is typically achieved using instruments such as ion sputtering. Considering cost and efficiency, it is advisable to design IVUS transducers in a specific way, dicing large assemblies into multiple smaller transducers after the electrodes have been led out. This approach enables the mass production of IVUS transducers and reduces costs.

The small size of high-frequency single-element IVUS transducers also determines that traditional saw dicing is not suitable for them. Except for dividing the components into individual ultrasound transducers (motor-driven) or for physically isolating multiple IVUS transducers of different frequencies [81], saws are usually not used in fabricating single-element IVUS transducers. Despite the fact that ultrasound transducers fabricated by saw cutting can achieve operating frequencies above 20 MHz, for side-looking phased-array IVUS transducers, the relatively wide pitches (>0.5 λ) make them unsuitable for intravascular applications, owning to the generation of spurious grating lobes, which prevents them from being used as phased-array IVUS with electronic beam steering [93]. However, this constraint does not apply uniformly: side-looking phased arrays with pitches >0.5λ remain viable for circumferential imaging where grating lobes are mitigated by limited imaging depth, tissue attenuation, and apodization. In contrast, forward-looking phased-array IVUS, which requires only ±30°–±45° electronic steering for chronic total occlusion navigation and device guidance, can tolerate wider pitches without severe grating lobe artifacts, as demonstrated by Cabrera–Munoz et al.’s 30 MHz, 32-element forward-looking array [87]. Thus, the suitability of phased-array IVUS depends critically on the imaging geometry: side-looking arrays prioritize circumferential coverage with fine angular sampling, while forward-looking designs sacrifice maximum resolution for real-time steering capability, with pitch constraints relaxed accordingly [94]. In addition to physically separating the array elements, electrical isolation between elements can also be achieved by patterning electrodes on the piezoelectric material plate, which are referred to as kerfless-arrayed transducers [56,95]; however, these transducers exhibit higher levels of crosstalk compared to their counterparts with kerfs [96]. Nevertheless, the resulting active apertures are small enough to be used as transducers in intravascular ultrasound catheters [94]. 

In recent years, with the development of semiconductor processes, etching processes represented by deep reactive ion etching (DRIE) have gradually been applied in the field of transducer manufacturing [97]. DRIE is an advanced microfabrication technology used for creating high aspect ratio micro- and nanostructures, and it is widely applied in the fields of Micro-Electro-Mechanical Systems (MEMS), Nano-Electro-Mechanical Systems (NEMS), three-dimensional integration of integrated circuits, and advanced packaging. In the field of transducers, DRIE is commonly used to manufacture 2-2 composite piezoelectric materials (alternating layers of piezoelectric and passive polymer phases arranged in two dimensions, resembling a layered sandwich structure) and 1-3 composite piezoelectric materials (piezoelectric pillars or posts embedded in a three-dimensional polymer matrix, where the “1” denotes the one-dimensional connectivity of the piezoelectric phase and the “3” denotes the three-dimensional connectivity of the polymer phase) with a smaller kerf width [98,99,100]. Based on DRIE technology, Cabrera-Munoz et al. [87] fabricated a forward-looking 30 MHz phased-array transducer for peripheral intravascular imaging by further etching the 2-2 composite single-crystal material, and encapsulated it within an 8 F catheter that is 0.6 m long. The resulting transducer exhibited an average center frequency of 28.9 MHz, an average bandwidth of 36.4% (−6 dB), and a crosstalk less than −26.5 dB. 

Crosstalk in ultrasound transducer arrays occurs through three primary pathways: mechanical/acoustic coupling via substrate modes and lateral waves, electrical coupling through cables and ground impedance, and electromechanical coupling through matching/backing layers [101]. For ICE arrays (5–10 MHz), typical crosstalk ranges from −24 dB to −33 dB, with DRIE-fabricated 1-3 PMN-PT composites achieving −33 dB isolation using 8 μm pillars with 4 μm kerfs [102]. IVUS arrays (20–60 MHz) face more severe challenges: kerfed arrays achieve −25 dB to −30 dB [103], while kerfless configurations suffer −11 dB to −15 dB due to a lack of physical separation [95]. Ma et al. [26] employed a 20 μm Al_2_O_3_/epoxy isolation layer in their 35/90 MHz dual-frequency IVUS transducer, achieving sufficient electrical isolation for multi-frequency operation; Liu et al. [102] utilized DRIE-fabricated pseudo-random 1–3 composites with <4 μm kerf widths, reducing crosstalk to −33 dB for 60 MHz IVUS arrays; and Wildes et al. [15] eliminated cable coupling entirely through ASIC integration in their 10 Fr 4D ICE catheter, though thermal management emerged as a secondary challenge.

The connection and isolation of acoustic and electrical signals determine the performance of ultrasonic transducers. As single-use, miniaturized transducers for ICE and IVUS catheters, the design must balance manufacturing costs and performance.

As a real-time imaging technology in interventional surgeries, the core value of intracardiac echocardiography ICE and IVUS lies in providing real-time intraoperative guidance. However, there are significant differences between these two technologies in terms of imaging range and operational costs compared to other techniques. Although coronary angiography is the traditional gold standard for interventional surgeries, it cannot assess the structural characteristics of the vascular wall (Table 5) [104,105,106].

The performance metrics in Table 1 correlate systematically with manufacturing constraints. High-frequency IVUS (greater than 40 MHz) requires microfabrication precision (kerf width less than 15 μm, matching layer thickness less than 20 μm) achievable only via deep reactive ion etching or precision dicing [97], elevating PMN-PT single crystals and 1–3 composites despite their higher cost. Conversely, ICE frequencies (5–10 MHz) tolerate conventional machining (kerf width greater than 50 μm), preserving PZT ceramics’ cost advantage. This frequency–precision–cost triad explains why material transitions (from PZT to PMN-PT) and process innovations (from dicing to etching to MEMS) occur heterogeneously across the frequency spectrum, rather than uniformly. The convergence point lies in the shared requirement for sub-millimeter element dimensions and multi-channel integration, driving both modalities toward ASIC-compatible fabrication and composite microarchitecture regardless of baseline frequency.

## 3. Conclusions and Perspectives

### 3.1. Functional Materials, Design Principles and Recent Advances in ICE/IVUS Micro-Transducers

In this paper, the functional materials used in ICE/IVUS applications, as well as the corresponding design principles and assembly processes, are explained. Additionally, some representative works in this field in recent years are briefly introduced, with the hope of providing assistance to those who are just entering the field of micro-transducer processes. The next-generation ICE and IVUS transducers will likely evolve along three convergent pathways. First, material hybridization (for example, PIN-PMN-PT single crystal 1–3 composites combining a high electromechanical coupling coefficient and designable impedance) enables bandwidth expansion beyond current limits. Second, monolithic integration (CMOS-compatible piezoelectric micromachined ultrasound transducers and capacitive micromachined ultrasound transducers with on-chip electronics) facilitates catheter miniaturization to sub-3 Fr profiles. Third, multi-physics fusion (IVUS-OCT-FFR co-registered catheters) provides diagnostic comprehensiveness for complex coronary interventions. Each pathway leverages distinct core competencies: composites for performance enhancement, MEMS for manufacturing scalability, and algorithmic co-registration for clinical utility. This suggests that technological convergence will occur predominantly at the system integration level rather than the material substitution level, with ICE and IVUS sharing composite microfabrication, ASIC integration, and heterogeneous packaging solutions across their respective development pipelines.

The design and manufacturing processes of micro-transducers always require continuous compromises to strike a balance between clinical performance and manufacturing complexity. Advanced piezoelectric materials—particularly PIN-PMN-PT single crystals and 1–3 composites—remain the current mainstream for ICE/IVUS transducers due to their mature manufacturing infrastructure and proven clinical performance. However, micromachined ultrasound transducers (MUTs) enabled by MEMS fabrication technology are emerging as promising alternatives. Capacitive micromachined ultrasonic transducers (CMUTs) offer fractional bandwidth exceeding 100% and monolithic integration with CMOS processes, facilitating catheter miniaturization and front–end electronics integration [107]; piezoelectric micromachined ultrasonic transducers (PMUTs) operate without DC bias and achieve monolithic CMOS integration through low-temperature processes (<400 °C), simplifying electronic system design [108]. These technologies specifically address the technical bottlenecks of bulk piezoelectric transducers: photolithography enables kerf widths of 3–4 μm, overcoming the precision limits of mechanical dicing for high-frequency IVUS (>40 MHz); meanwhile, MUT integration with application-specific integrated circuits (ASICs) substantially reduces cable counts for high-density arrays, resolving the wiring challenges of real-time 3D ICE imaging [109]. The future trend points toward convergent innovation—where piezoelectric transducers and MEMS technologies will complement each other across different clinical applications rather than simple substitution.

With the increasing global aging population, the demand for ICE/IVUS catheters in the clinical diagnosis of cardiovascular diseases will continue to rise. Real-time 3D (4D) imaging catheters will become more widespread. Meanwhile, the integration of catheters with other diagnostic methods and therapeutic techniques, such as optical coherence tomography (OCT), fractional flow reserve (FFR), electrophysiology, and surgical robotic technologies, will provide more comprehensive diagnostic information for cardiovascular diseases and simplify surgical procedures, thereby reducing patient suffering. This trend demands that ICE/IVUS transducers achieve further miniaturization while maintaining higher imaging performance, in order to be packaged into smaller-bore multi-lumen catheters. Undoubtedly, this will pose new challenges for the design and manufacturing processes of the transducers.

The operation of ICE and IVUS demands proficiency in both catheter manipulation and image interpretation. Training programs typically progress from equipment fundamentals to simulated procedures and supervised clinical practice, with competency benchmarks varying across institutions [110]. Learning curve studies indicate that operators generally require 20–50 supervised cases to achieve technical proficiency for standard procedures [111], with complex interventions such as structural heart disease treatments necessitating additional specialized training [112].

The clinical adoption of ICE and IVUS technologies is influenced by economic factors, including device procurement costs and procedural reimbursement policies. Cost-structure analyses indicate that transducer materials (piezoelectric crystals and composites) and catheter manufacturing processes constitute the primary cost drivers, collectively accounting for approximately 60–70% of disposable catheter expenses [113]. Lead-free piezoelectric alternatives such as BZT-50BCT have demonstrated potential for material cost reduction upon scale-up [42], though performance trade-offs remain a consideration for high-frequency applications. Manufacturing automation represents another avenue for cost optimization, with studies suggesting that standardized packaging processes could improve production efficiency [114].

The accessibility enhancement strategy encompasses two key components: tiered application and medical insurance policy adjustments. Regarding tiered application, primary care hospitals will be prioritized for basic IVUS (35 MHz) configurations for routine PCI procedures, while tertiary hospitals will adopt 4D ICE combined with dual-frequency IVUS for complex surgeries. In terms of medical insurance policies, ICE/IVUS devices will be included in high-value consumables centralized procurement programs (such as China’s coronary stent procurement model), with prices expected to decrease by 50% compared to current levels [115].

### 3.2. Potential Optimization Directions for 4D ICE Real-Time 3D Reconstruction

Research directions for advancing 4D ICE technology have focused on enhancing imaging frame rates. Studies have explored increasing frame rates from 30 to 60 frames per second to improve real-time guidance during arrhythmia procedures [116]. Technical approaches under investigation include the application of high-bandwidth materials such as PIN-PMN-PT single-crystal 1–3 composites to reduce signal acquisition duration, and the integration of ASIC chips for multi-channel signal processing to enhance data throughput [90,91,92]. Thermal management remains a critical engineering constraint, with ASIC heat dissipation requiring materials capable of maintaining intracardiac temperatures within safe physiological limits, prompting research into ceramic-based thermal management solutions with thermal conductivity values above 20 W/(m·K) [116].

### 3.3. Integrated IVUS and OCT Catheter

Integrated IVUS-OCT systems aim to combine IVUS-derived vascular wall structure information with OCT-based plaque microstructure characterization, with reported spatial resolutions of approximately 2 mm for IVUS and 10 μm for OCT, respectively [117]. Technical implementations have employed coaxial catheter designs integrating an IVUS transducer with surrounding OCT fiber bundles, achieving catheter diameters of 3.5 Fr. Image co-registration techniques have demonstrated alignment errors below 0.1 mm, enabling complementary assessment of calcified plaques via IVUS and lipid cores via OCT. However, OCT imaging sensitivity to blood interference necessitates continuous saline irrigation, contributing to procedural complexity and increased consumable costs relative to standalone IVUS catheters [118].

Demographic trends, including population aging, have been associated with increasing clinical utilization of ICE and IVUS technologies [119]. The evolution of interventional cardiology has seen growing adoption of 4D (real-time 3D) imaging modalities, with market analyses indicating expanded penetration of volumetric imaging catheters [118]. Convergence with complementary diagnostic technologies—such as OCT, FFR measurement, electrophysiological mapping, and surgical robotic systems—has been explored to enhance diagnostic comprehensiveness and procedural efficiency [27,118,120]. These developments impose evolving material requirements, including enhanced thermomechanical stability, improved electromechanical conversion efficiency, and miniaturization, to accommodate multi-lumen catheter designs with sub-millimeter profiles [121]. Undoubtedly, this process will present significant challenges to catheter design and manufacturing processes.

Furthermore, with the development of novel biocompatible materials and matching layers along with backing materials, these innovations not only deliver superior electrical and acoustic performance but also reduce bonding and cutting costs, enabling more idealized designs for electroacoustic transducers [102]. Moreover, as microelectromechanical system (MEMS) technology continues to advance, manufacturing processes for ultrasonic transducers will undergo continuous refinement, propelling ICE/IVUS technologies to new heights.

## Figures and Tables

**Table 1:** Performance comparison of ICE and IVUS transducers based on different piezoelectric materials.

Ref.	Material Class	Center Frequency (MHz)	Bandwidth (%)	Axial Resolution (μm)	Lateral Resolution (μm)
[14]	PZT Ceramic	6.2	61	Not reported	Not reported
[15]	PZT Ceramic	5.6	60	Not reported	Not reported
[33]	PMN-PT Single crystal	82	65	35	176
[34]	PMN-PT Single crystal	47	72	25	120
[35]	PMN-PT Single crystal	46.8	67.2	40	210
[39]	1–3 Composite	9	55	108.5	317
[40]	1–3 PIN-PMN-PT	41	86	43	226
[45]	1–3 PZT Composite	51.2	68.8	22	Not reported
[41]	1–3 PMN-PT	41	77	Not reported	Not reported
[42]	BZT-50BCT Lead-free	30.5	53	Not reported	Not reported
[44]	KNN Lead-free	52	61.5	Not reported	Not reported
[29]	PMN-PT Single crystal	35	47.7	46.0	231.5
[29]	PMN-PT Single crystal	90	42.0	21.5	123.5
[29]	LiNbO_3_ Single crystal	120	24.1	25.7	105.3
[29]	LiNbO_3_ Single crystal	150	28.1	17.2	87.3
[46]	PMN-32%PT	60.2	57.2	24.8	156.1

Notes: bandwidth: −6 dB fractional bandwidth measured by two-way pulse-echo method in water at 20–25 °C. The transducer was excited by a spike pulse (Panametrics 5900PR or equivalent), and the echo from a plane reflector (stainless steel or glass, positioned at focal distance or near-field/far-field transition) was received and analyzed by fast Fourier transform (FFT). The −6 dB points were identified at 50% of the peak spectral amplitude, and fractional bandwidth was calculated as 2(*f_H_* − *f_L_*)/(*f_H_* + *f_L_*) × 100%, where f_H_ and f_L_ are the upper and lower −6 dB frequencies, and f_c_ = (*f_H_* + *f_L_*)/2 is the center frequency [47]. Lateral resolution: −6 dB beam width at focal point, measured by scanning a line target or from beam profile analysis; Axial resolution: −6 dB pulse width in water, calculated from the echo waveform.

**Table 2:** Commercial catheter sheath candidates for the ICE/IVUS catheter and their acoustic properties.

Ref.	Materials	Density ρ (g/cm^3^)	Longitudinal Velocity V_l_ (m/s)	Acoustic Impedance Z (MRayl)	Attenuation Coefficient α (dB/cm@1 MHz)	Frequency Exponent β	Test Conditions
	Biological Tissues						
[50]	Cardiac Muscle	1.06	1595	1.69	0.52	—	37 °C
[50]	Blood	1.06	1590	1.68	0.16	—	37 °C
	Polyether Block Amide (PEBA) Copolymers						
[49]	Pebax 2533 (25D)	1.01	1520	1.54	1.12	1.40	37 °C, pulse-echo
[49]	Pebax 3533 (35D)	1.01	1529	1.55	1.54	1.28	37 °C, pulse-echo
[49]	Pebax 4033 (40D)	1.01	1657	1.67	2.67	1.21	37 °C, pulse-echo
[49]	Pebax 5533 (55D)	1.01	1751	1.77	3.41	1.24	37 °C, pulse-echo
[49]	Pebax 6333 (63D)	1.01	1881	1.90	3.35	1.27	37 °C, pulse-echo
[49]	Pebax 7033 (70D)	1.01	1993	2.01	2.39	1.25	37 °C, pulse-echo
	Aromatic Polyurethanes						
[49]	Pellethane 2363 (80A)	1.13	1648	1.86	6.23	1.19	37 °C, pulse-echo
[49]	Pellethane 2363 (90A)	1.14	1779	2.03	8.60	1.05	37 °C, pulse-echo
[49]	Pellethane 2363 (75D)	1.21	1955	2.37	4.36	1.15	37 °C, pulse-echo
	Aliphatic Polyurethanes						
[49]	Tecoflex (80A)	1.04	1524	1.59	1.73	1.47	37 °C, pulse-echo
[49]	Tecoflex (65D)	1.10	1951	2.15	4.58	1.17	37 °C, pulse-echo
	Two-Part Thermoset Polyurethanes						
[51]	RP 6400	1.04	1480 (37 °C)/1540 (25 °C)	1.54	10	~1.0 †	37 °C or 25 °C, method not specified
[51]	Bacon 430	1.10	1746 (37 °C)/1870 (25 °C)	1.92	7.7	~1.0 †	37 °C or 25 °C, method not specified
[51]	Santoprene	0.97	1474 (37 °C)/1535 (25 °C)	1.43	5	~1.0 †	37 °C or 25 °C, method not specified
	Other Polymers						
[52]	TPX (Polymethylpentene)	0.83	2190	1.82	1.2	—	Temperature not specified

Notes: Frequency dependence of attenuation: the attenuation coefficient follows the power-law relationship: α(f) = α_0_(f/f_0_)^β, where f_0_ = 1 MHz. The exponent β is provided in the table where available. For polyurethane materials, β typically ranges from 1.0 to 1.5. † Estimated value; frequency dependence not explicitly reported in original reference.

**Table 3:** Insertion loss and bandwidth performance of various transducer matching layer strategies.

Ref.	Matching Strategy	1st Layer Z (MRayl)	2nd Layer Z (MRayl)	Center Frequency (MHz)	Insertion Loss (dB)	−6 dB Bandwidth (%)	Fabrication Complexity
[42]	Ag-epoxy + Parylene	~7.33	~2.59	30.5	18.7 (measured)	53	Low
[14,15]	Metal-filled Graphite + ABS	~6.6	~2.2	~6	Not reported	60–61	Low
[71]	Ag-epoxy + Parylene	7.33	2.59	50.84	Not reported	68.48%	Medium
[72]	Single-layer E-solder	5.9	None	43.5	Not reported	62.3	Low
[73]	Parylene only (>100 MHz)	2.59	None	120	Not reported	24.1	Low
[26]	Mass-Spring +Parylene C	Variable	Variable	47	−21.9	46	Very High
[75]	FPC-based	Variable (Cu/PI)	Variable	Variable	Not reported	—	Medium

Notes: insertion loss: “measured” indicates direct experimental measurement, two-way insertion loss measured in water using the pulse-echo response from a plane reflector at a focal distance with a broadband receiver; other values estimated from reported electrical impedance and sensitivity data. Fabrication complexity: “Low” = castable epoxy with grinding; “Medium” = vapor deposition or FPC-based; “Very High” = photolithography/etching or mass-spring structure. Impedance values for composite matching layers calculated from volume fraction and constituent properties.

**Table 4:** Catheter outer diameter, array channel count, and cable strategy configurations for ICE and IVUS transducers in the literature.

Device Type	Catheter OD (Fr)	Channel Count	Cable Strategy	Center Frequency (MHz)	Cable Loss @ Freq	Key Challenge
4D ICE Motor-Driven [14]	10	N/A (Motor)	Motor/Coaxial	5–10	Not critical (low frequency)	Motor reliability/Hermeticity
4D ICE Phased Array [15]	10	64–128	ASIC-integrated	5–10	Eliminated (on-chip)	ASIC heat dissipation (37 °C limit)
Standard ICE (Commercial)	6–10	32–64	Standard micro-coax	5–10	Low (<0.5 dB)	Standard design
IVUS Mechanical [23]	3–6	N/A (Motor)	Motor/Coaxial	20–60	~0.61 dB/m @ 35 MHz	NURD/Off-axis errors
IVUS Phased Array [11]	3.5–9	32–64	Micro-coax bundle	20–60	Critical (>1 dB @ 60 MHz)	Element isolation/Crosstalk
IVUS Dual Frequency [29]	3–6	2 (Back-to-back)	Shared backing	35/90	Critical (impedance mismatch)	Signal isolation between frequencies
IVUS Forward-Looking [87]	8	32	Micro-coax bundle	30	Moderate	Fabrication complexity
IVUS Phased Array [56]	3.5–9	32–64	Micro-coax	35	0.61 dB/m @ 35 MHz	Cable loading at high frequency
4D ICE Phased Array [88]	10	1024 (32 × 32)	ASIC-integrated	5–10	Eliminated (ASIC)	ASIC thermal management
IVUS Dual Frequency [25]	3	2 (Dual-freq)	Shared backing	35/90	Minimized (short distance)	Miniaturization limit

**Table 5:** Comparison of different imaging techniques in cardiovascular diagnosis.

The Dimension of Comparison	Imaging Scenario	Resolution Ratio	Real-Time	Operating Risk	Core Clinical Value	Limitations	Single Operation Cost
ICE	Intracardiac structures (atrium, valves, endocardium)	High (intracardiac structure, 10–50 μm)	Real-time (30–60 frames/s, 4D ICE supports dynamic reconstruction)	Low (via vascular intervention, no radiation, no general anesthesia required)	Real-time intracardiac intervention positioning (e.g., release of a stent)	Field of view is limited (only in the cardiac cavity)	Higher (the catheter is disposable, about 10,000–20,000 CNY)
IVUS	Intravascular structures (coronary wall, plaque, stent)	Extremely high (microstructure of blood vessel wall, 5–20 μm)	Real-time (15–30 frames/s, pullback imaging)	Low (via coronary intervention, no radiation)	Coronary intervention plaque/stent assessment	Field of view limitation (only in the vascular lumen, near field blind area)	Higher (the catheter is disposable, about 8000–15,000 CNY)
TTE/TEE	Whole heart structure (trans-thoracic/trans-esophageal)	Central overall structure (100–500 μm)	Real-time (20–30 frames/s)	TEE requires general anesthesia (risk of esophageal injury)	Preoperative structure evaluation and postoperative efficacy follow-up	TTE is interfered by lung gas/obesity, and TEE cannot be used in operation	Low (device reuse, about 10,000–30,000 CNY)
CT/MRI	Preoperative anatomical evaluation (vascular and cardiac morphology	High (three-dimensional anatomy, 50–200 μm)	Non-real-time (needs to be reconstructed later)	Exposure to radiation (CT), requires contrast agent (kidney injury risk)	Preoperative complex structural anatomy planning	Unable to guide intervention in real time	Medium (CT about 3000–5000 CNY, MRI about 8000 CNY)
CAG	Assessment of lumen stenosis	Low (shows the filling range of contrast agent)	Real-time (contrast flow observation)	Radiation/contrast agent allergy	Preliminary judgment of coronary stenosis	It is impossible to assess the nature of the vascular wall and plaque	Medium (about 5000–8000 CNY)

## Data Availability

No new data were created or analyzed in this study. Data sharing is not applicable to this article.

## References

[1] Alqahtani F., Bhirud A., Aljohani S., Mills J., Kawsara A., Runkana A., Alkhouli M. (2017). Intracardiac versus transesophageal echocardiography to guide transcatheter closure of interatrial communications: Nationwide trend and comparative analysis. J. Interv. Cardiol..

[2] Mojadidi M.K., Gevorgyan R., Tobis J.M., Amin Z., Tobis J.M., Sievert H., Carroll J.D. (2015). A Comparison of Methods to Detect and Quantitate PFO: TCD, TTE, ICE and TEE. Patent Foramen Ovale.

[3] Lee S.H., Oh S., Ko Y.G., Lee Y.J., Lee S.J., Hong S.J., Ahn C.M., Kim J.S., Kim B.K., Ko K.Y. (2024). Comparison of intracardiac echocardiography versus transesophageal echocardiography for guidance during transcatheter aortic valve replacement. Korean Circ. J..

[4] Mathur S.K., Singh P. (2009). Transoesophageal echocardiography related complications. Indian. J. Anaesth..

[5] Jongbloed M.R., Schalij M.J., Zeppenfeld K., Oemrawsingh P.V., van der Wall E.E., Bax J.J. (2005). Clinical applications of intracardiac echocardiography in interventional procedures. Heart.

[6] Freitas-Ferraz A.B., Bernier M., Vaillancourt R., Ugalde P.A., Nicodème F., Paradis J.-M., Champagne J., O’Hara G., Junquera L., Val D.d. (2020). Safety of Transesophageal Echocardiography to Guide Structural Cardiac Interventions. J. Am. Coll. Cardiol..

[7] Saito Y., Kobayashi Y., Fujii K., Sonoda S., Tsujita K., Hibi K., Morino Y., Okura H., Ikari Y., Kozuma K. (2025). CVIT 2025 clinical expert consensus document on intravascular ultrasound. Cardiovasc. Interv. Ther..

[8] Räber L., Mintz G.S., Koskinas K.C., Johnson T.W., Holm N.R., Onuma Y., Radu M.D., Joner M., Yu B., Jia H. (2018). Clinical use of intracoronary imaging. Part 1: Guidance and optimization of coronary interventions. An expert consensus document of the European Association of Percutaneous Cardiovascular Interventions. Eur. Heart J..

[9] Jeger R.V., Eccleshall S., Wan Ahmad W.A., Ge J., Poerner T.C., Shin E.S., Alfonso F., Latib A., Ong P.J., Rissanen T.T. (2020). Drug-Coated Balloons for Coronary Artery Disease: Third Report of the International DCB Consensus Group. JACC Cardiovasc. Interv..

[10] Gao X.F., Ge Z., Kong X.Q., Chen X., Han L., Qian X.S., Zuo G.F., Wang Z.M., Wang J., Song J.X. (2024). Intravascular Ultrasound vs Angiography-Guided Drug-Coated Balloon Angioplasty: The ULTIMATE III Trial. JACC Cardiovasc. Interv..

[11] Peng C., Wu H., Kim S., Dai X., Jiang X. (2021). Recent Advances in Transducers for Intravascular Ultrasound (IVUS) Imaging. Sensors.

[12] Sung J.-H., Chang J.-H. (2021). Mechanically rotating intravascular ultrasound (IVUS) transducer: A review. Sensors.

[13] Leuzzi F., Formisano C., Cerrato E., Maione A., Attisano T., Meucci F., Ciccarelli M., Vecchione C., Galasso G., Di Muro F.M. (2026). Intracardiac Echocardiography in Structural Heart Interventions: A Comprehensive Overview. J. Clin. Med..

[14] Lee W., Griffin W., Wildes D., Buckley D., Topka T., Chodakauskas T., Langer M., Calisti S., Bergstøl S., Malacrida J.P. (2011). A 10-Fr ultrasound catheter with integrated micromotor for 4-D intracardiac echocardiography. IEEE Trans. Ultrason. Ferroelectr. Freq. Control.

[15] Wildes D., Lee W., Haider B., Cogan S., Sundaresan K., Mills D.M., Yetter C., Hart P.H., Haun C.R., Concepcion M. (2016). 4-D ICE: A 2-D Array Transducer With Integrated ASIC in a 10-Fr Catheter for Real-Time 3-D Intracardiac Echocardiography. IEEE Trans. Ultrason. Ferroelectr. Freq. Control.

[16] Tang G.H.L., Zaid S., Hahn R.T., Aggarwal V., Alkhouli M., Aman E., Berti S., Chandrashekhar Y.S., Chadderdon S.M., D’Agostino A. (2025). Structural Heart Imaging Using 3-Dimensional Intracardiac Echocardiography. JACC Cardiovasc. Imaging.

[17] Khalili H., Patton M., Taii H.A., Bansal P., Brady M., Taylor J., Gurung A., Maini B. (2019). 4D Volume Intracardiac Echocardiography for Intraprocedural Guidance of Transcatheter Left Atrial Appendage Closure. J. Atr. Fibrillation.

[18] Pommier T., Guenancia C., Sagnard A., Ferrand B., Didier R., Fichot M., Laurent G., Morgant M.C., Bouchot O., Lorgis L. (2021). Safety and Efficacy of Transcatheter Mitral Valve Replacement Guided by Intracardiac Echocardiography. JACC Cardiovasc. Interv..

[19] Bhardwaj B., Lantz G., Golwala H., Chadderdon S., Song H.K., Zahr F. (2022). Transcatheter Valve-in-Valve Mitral Valve Replacement Using 4D Intracardiac Echocardiogram and Conscious Sedation. Struct. Heart.

[20] Bartel T., Bonaros N., Müller L., Friedrich G., Grimm M., Velik-Salchner C., Feuchtner G., Pedross F., Müller S. (2011). Intracardiac echocardiography: A new guiding tool for transcatheter aortic valve replacement. J. Am. Soc. Echocardiogr..

[21] Ussia G.P., Barbanti M., Sarkar K., Cumbo M., Aruta P., Scarabelli M., Cammalleri V., Immè S., Pistritto A.M., Gulino S. (2012). Accuracy of intracardiac echocardiography for aortic root assessment in patients undergoing transcatheter aortic valve implantation. Am. Heart J..

[22] Nielsen-Kudsk J.E., Berti S., Caprioglio F., Ronco F., Arzamendi D., Betts T., Tondo C., Christen T., Allocco J. (2023). Intracardiac echocardiography to guide Watchman FLX implantation: The ICE LAA study. Cardiovasc. Interv..

[23] Cabrera-Munoz N.E., Eliahoo P., Wodnicki R., Jung H., Chiu C.T., Williams J.A., Kim H.H., Zhou Q., Yang G.Z., Shung K.K. (2019). Fabrication and Characterization of a Miniaturized 15-MHz Side-Looking Phased-Array Transducer Catheter. IEEE Trans. Ultrason. Ferroelectr. Freq. Control.

[24] Shlofmitz E., Kerndt C.C., Parekh A., Khalid N. (2023). Intravascular ultrasound. StatPearls.

[25] Munding C.E., Chérin E., Jourard I., Weyers J.J., Goertz D.E., Courtney B.K., Foster F.S. (2018). Development of a 3 French dual-frequency intravascular ultrasound catheter. Ultrasound Med. Biol..

[26] Ma T., Yu M., Li J., Munding C.E., Chen Z., Fei C., Shung K.K., Zhou Q. (2015). Multi-frequency intravascular ultrasound (IVUS) imaging. IEEE Trans. Ultrason. Ferroelectr. Freq. Control.

[27] Ono M., Kawashima H., Hara H., Gao C., Wang R., Kogame N., Takahashi K., Chichareon P., Modolo R., Tomaniak M. (2020). Advances in IVUS/OCT and future clinical perspective of novel hybrid catheter system in coronary imaging. Front. Cardiovasc. Med..

[28] Ma J., Martin K., Dayton P.A., Jiang X. (2014). A preliminary engineering design of intravascular dual-frequency transducers for contrast-enhanced acoustic angiography and molecular imaging. IEEE Trans. Ultrason. Ferroelectr. Freq. Control.

[29] Ma J., Martin K.H., Li Y., Dayton P.A., Shung K.K., Zhou Q., Jiang X. (2015). Design factors of intravascular dual frequency transducers for super-harmonic contrast imaging and acoustic angiography. Phys. Med. Biol..

[30] Wang Z., Heath Martin K., Huang W., Dayton P.A., Jiang X. (2017). Contrast Enhanced Superharmonic Imaging for Acoustic Angiography Using Reduced Form-Factor Lateral Mode Transmitters for Intravascular and Intracavity Applications. IEEE Trans. Ultrason. Ferroelectr. Freq. Control.

[31] Maresca D., Renaud G., Soest G., Li X., Zhou Q.F., Shung K.K., Jong N., Steen A.F.W. (2013). Contrast-enhanced intravascular ultrasound pulse sequences for bandwidth-limited transducers. Ultrasound Med. Biol..

[32] Li Y., Ma J., Martin K.H., Yu M., Ma T., Dayton P.A., Jiang X., Shung K.K., Zhou Q. (2016). An Integrated System for Superharmonic Contrast-Enhanced Ultrasound Imaging: Design and Intravascular Phantom Imaging Study. IEEE Trans. Biomed. Eng..

[33] Li X., Wu W., Chung Y., Shih W.Y., Shih W.H., Zhou Q., Shung K.K. (2011). 80-MHz intravascular ultrasound transducer using PMN-PT free-standing film. IEEE Trans. Ultrason. Ferroelectr. Freq. Control.

[34] Yoon S., Williams J., Kang B.J., Yoon C., Cabrera-Munoz N., Jeong J.S., Lee S.G., Shung K.K., Kim H.H. (2015). Angled-focused 45 MHz PMN-PT single element transducer for intravascular ultrasound imaging. Sens. Actuators A Phys..

[35] Ma X., Cao W. (2020). Single-Crystal High-Frequency Intravascular Ultrasound Transducer With 40-μm Axial Resolution. IEEE Trans. Ultrason. Ferroelectr. Freq. Control.

[36] Yuan J., Jiang X., Snook K., Rehrig P., Shrout T., Hackenberger W.S., Cheng A., Cao P., Lavalelle G., Geng X. (2006). 5I-1 Microfabrication of piezoelectric composite ultrasound transducers (PC-MUT). Proceedings of the 2006 IEEE Ultrasonics Symposium.

[37] Shung K.K. (2015). Diagnostic Ultrasound: Imaging and Blood Flow Measurements.

[38] Steinhausen R., Pientschke C., Kern S., Beige H. (2011). A8. 4-Modelling and Characterization of Piezoelectric 1–3 Fiber Composites. Proc. Sens..

[39] Han Z., Wang N., Li Z., Zhu X., Chen Y., Jian X., Cui Y. (2021). Phased-array transducer for intracardiac echocardiography based on 1–3 piezocomposite. Front. Mater..

[40] Li X., Ma T., Tian J., Han P., Zhou Q., Shung K.K. (2014). Micromachined PIN-PMN-PT crystal composite transducer for high-frequency intravascular ultrasound (IVUS) imaging. IEEE Trans. Ultrason. Ferroelectr. Freq. Control.

[41] Yuan J.R., Jiang X., Cao P.J., Sadaka A., Bautista R., Snook K., Rehrig P.W. 5C-5 High Frequency Piezo Composites Microfabricated Ultrasound Transducers for Intravascular Imaging (Invited). Proceedings of the 2006 IEEE Ultrasonics Symposium.

[42] Yan X., Lam K.H., Li X., Chen R., Ren W., Ren X., Zhou Q., Shung K.K. (2013). Lead-free intravascular ultrasound transducer using BZT-50BCT ceramics. IEEE Trans. Ultrason. Ferroelectr. Freq. Control.

[43] Liu W., Ren X. (2009). Large piezoelectric effect in Pb-free ceramics. Phys. Rev. Lett..

[44] Zhu B., Zhang Z., Ma T., Yang X., Li Y., Shung K.K., Zhou Q. (2015). (100)-Textured KNN-based thick film with enhanced piezoelectric property for intravascular ultrasound imaging. Appl. Phys. Lett..

[45] Li Z., Lv J., Zhu X., Cui Y., Jian X. (2021). Development of high frequency piezocomposite with hexagonal pillars via cold ablation process. Ultrasonics.

[46] Sung J.H., Jeong J.S. (2018). Development of High-Frequency (>60 MHz) Intravascular Ultrasound (IVUS) Transducer by Using Asymmetric Electrodes for Improved Beam Profile. Sensors.

[47] Caronti A., Caliano G., Carotenuto R., Savoia A., Pappalardo M., Cianci E., Foglietti V. (2006). Capacitive micromachined ultrasonic transducer (CMUT) arrays for medical imaging. Microelectron. J..

[48] Szabo T.L., Szabo T.L. (2014). Chapter 5—Transducers. Diagnostic Ultrasound Imaging: Inside Out.

[49] Guess J.F., Campbell J.S. (1995). Acoustic properties of some biocompatible polymers at body temperature. Ultrasound Med. Biol..

[50] O’Donnell M., Mimbs J., Miller J. (1979). The relationship between collagen and ultrasonic attenuation in myocardial tissue. J. Acoust. Soc. Am..

[51] Stephens D.N., Cannata J., Liu R., Zhao J.Z., Shung K.K., Nguyen H., Chia R., Dentinger A., Wildes D., Thomenius K.E. (2008). The acoustic lens design and in vivo use of a multifunctional catheter combining intracardiac ultrasound imaging and electrophysiology sensing. IEEE Trans. Ultrason. Ferroelectr. Freq. Control.

[52] Bloomfield P.E., Lo W.-J., Lewin P.A. (2000). Experimental study of the acoustical properties of polymers utilized to construct PVDF ultrasonic transducers and the acousto-electric properties of PVDF and P (VDF/TrFE) films. IEEE Trans. Ultrason. Ferroelectr. Freq. Control.

[53] Nimoh D., Acquah I., Wordui E. (2025). Rational Design of Alternative Natural-Based Coupling Media for Diagnostic Ultrasound Imaging: A Review. Biomed. Phys. Eng. Express.

[54] Bruining N., Hamers R., Teo T.-J., de Feijter P.J., Serruys P.W., Roelandt J.R. (2004). Adjustment method for mechanical Boston scientific corporation 30 MHz intravascular ultrasound catheters connected to a Clearview^®^ console. Int. J. Cardiovasc. Imaging.

[55] Corl P.D. (2013). Device and System for Imaging and Blood Flow Velocity Measurement. U.S. Patent.

[56] Zhu B., Chan N.Y., Dai J., Shung K.K., Takeuchi S., Zhou Q. (2013). New fabrication of high-frequency (100-MHz) ultrasound PZT film kerfless linear array [Correspondence]. IEEE Trans. Ultrason. Ferroelectr. Freq. Control.

[57] Cannata J.M., Williams J.A., Qifa Z., Ritter T.A., Shung K.K. (2006). Development of a 35-MHz piezo-composite ultrasound array for medical imaging. IEEE Trans. Ultrason. Ferroelectr. Freq. Control.

[58] Jian X., Han Z., Liu P., Xu J., Li Z., Li P., Shao W., Cui Y. (2017). A High Frequency Geometric Focusing Transducer Based on 1-3 Piezocomposite for Intravascular Ultrasound Imaging. BioMed Res. Int..

[59] Thangaraju P., Varthya S.B. (2022). ISO 10993: Biological evaluation of medical devices. Medical Device Guidelines and Regulations Handbook.

[60] Busch J.D., Schröder H., Sellenschloh K., Adam G., Ittrich H., Huber G. (2018). Test method for mechanical properties of implantable catheters according to DIN 10555-3. J. Mech. Behav. Biomed. Mater..

[61] Gulino M.-S., Bruzzi M., Vangi D. (2021). Gas-coupled laser acoustic detection technique for NDT of mechanical components. Ultrasonics.

[62] Basaeri H., Christensen D.B., Roundy S. (2016). A review of acoustic power transfer for bio-medical implants. Smart Mater. Struct..

[63] Tran-Huu-Hue L., Desmare R., Lavassort F., Lethiecq M. (1997). KLM circuit-based method for modeling multilayer piezoelectric structures. Proceedings of the 1997 IEEE Ultrasonics Symposium Proceedings. An International Symposium (Cat. No. 97CH36118).

[64] Desilets C.S., Fraser J.D., Kino G.S. (2005). The design of efficient broad-band piezoelectric transducers. IEEE Trans. Sonics Ultrason..

[65] Inoue T., Ohta M., Takahashi S. (1987). Design of ultrasonic transducers with multiple acoustic matching layers for medical application. IEEE Trans. Ultrason. Ferroelectr. Freq. Control.

[66] Yang G., Chen Y., Li M., Yang J., Xi S. (2025). Design of a Cylindrical Megahertz Miniature Ultrasonic Welding Oscillator. Sensors.

[67] Henneberg J., Gerlach A., Storck H., Cebulla H., Marburg S. (2018). Reducing mechanical cross-coupling in phased array transducers using stop band material as backing. J. Sound Vib..

[68] Ayter S. (1990). Transmission line modelling for array transducer elements. Proceedings of the IEEE Symposium on Ultrasonics.

[69] Arakawa T., Tateishi Y. Ceramic-Based Acoustic Matching Layer Material, Production Method Therefor, and Use Therefor. https://worldwide.espacenet.com/publicationDetails/biblio?DB=&ND=5&locale=en_EP&FT=D&date=20231005&CC=JP&NR=WO2023190097A1&KC=A1.

[70] Zhou Q., Cha J.H., Huang Y., Zhang R., Cao W., Shung K.K. (2009). Alumina/epoxy nanocomposite matching layers for high-frequency ultrasound transducer application. IEEE Trans. Ultrason. Ferroelectr. Freq. Control.

[71] Zhang Z., An X., Guo S., Gong X., Ke Q. (2023). Design and Fabrication of Annular-Array Ultrasound Transducer Based (K, Na) NbO 3 Lead-Free 1–3 Piezoelectric Composite. IEEE Trans. Ultrason. Ferroelectr. Freq. Control.

[72] Lee J., Jang J., Chang J.H. (2016). Oblong-shaped-focused transducers for intravascular ultrasound imaging. IEEE Trans. Biomed. Eng..

[73] Quan Y., Yang X., Fei C., Zhao T., Zhang J., Li Z., Sun X., Chen Q., Chen J., Yang Y. (2024). PZN-PT single crystal based high-frequency intravascular ultrasound transducers. Ceram. Int..

[74] Brown J.A., Sharma S., Leadbetter J., Cochran S., Adamson R. (2014). Mass-spring matching layers for high-frequency ultrasound transducers: A new technique using vacuum deposition. IEEE Trans. Ultrason. Ferroelectr. Freq. Control.

[75] Lee T., Jung J., Lee S.-M., Park J., Park J.-H., Paik K.-W., Lee H.J. (2022). FPCB as an acoustic matching layer for 1D linear ultrasound transducer arrays. Sensors.

[76] Hou C., Li Z., Fei C., Wei X., Yang Y., Wang Y., Quan Y., Yang Y. (2024). Optimal design of ultrasonic transducer based on multi-layer backing with adjustable impedance. Ceram. Int..

[77] Lange J.N. (1964). A Study of Ultrasonic Attenuation and Wave Propagation in Solids.

[78] Grewe M.G., Gururaja T., Shrout T.R., Newnham R.E. (2002). Acoustic properties of particle/polymer composites for ultrasonic transducer backing applications. IEEE Trans. Ultrason. Ferroelectr. Freq. Control.

[79] Kim H., Yoo J., Heo D., Seo Y.-S., Lim H.G., Kim H.H. (2022). High-attenuation backing layer for miniaturized ultrasound imaging transducer. IEEE Trans. Ultrason. Ferroelectr. Freq. Control.

[80] Qiu W., Chen Y., Li X., Yu Y., Cheng W.F., Tsang F.K., Zhou Q., Shung K.K., Dai J., Sun L. (2012). An open system for intravascular ultrasound imaging. IEEE Trans. Ultrason. Ferroelectr. Freq. Control.

[81] Lee J., Moon J.Y., Chang J.H. (2018). A 35 MHz/105 MHz Dual-Element Focused Transducer for Intravascular Ultrasound Tissue Imaging Using the Third Harmonic. Sensors.

[82] He Y., Liu X., Zhang J., Peng C. (2023). A Backing-Layer-Shared Miniature Dual-Frequency Ultrasound Probe for Intravascular Ultrasound Imaging: In Vitro and Ex Vivo Validations. Biosensors.

[83] Yang H.C., Yin J., Hu C., Cannata J., Zhou Q., Zhang J., Chen Z., Shung K.K. (2010). A dual-modality probe utilizing intravascular ultrasound and optical coherence tomography for intravascular imaging applications. IEEE Trans. Ultrason. Ferroelectr. Freq. Control.

[84] Lee J., Chang J.H. (2018). A 40-MHz Ultrasound Transducer with an Angled Aperture for Guiding Percutaneous Revascularization of Chronic Total Occlusion: A Feasibility Study. Sensors.

[85] Liu X., Li Y., Qin H., Peng C. (2025). High-Frequency 64-Element Ring-Annular Array Transducer: Development and Preclinical Validation for Intravascular Ultrasound Imaging. Biosensors.

[86] Kilroy J.P., Patil A.V., Rychak J.J., Hossack J.A. (2014). An IVUS transducer for microbubble therapies. IEEE Trans. Ultrason. Ferroelectr. Freq. Control.

[87] Cabrera-Munoz N.E., Eliahoo P., Wodnicki R., Jung H., Chiu C.T., Williams J.A., Kim H.H., Zhou Q., Shung K.K. (2018). Forward-looking 30-MHz phased-array transducer for peripheral intravascular imaging. Sens. Actuators A: Phys..

[88] Dos Santos D.S., Ossenkoppele B., Hopf Y.M., Soozande M., Noothout E., Vos H.J., Bosch J.G., Pertijs M.A., Verweij M.D., de Jong N. (2024). An ultrasound matrix transducer for high-frame-rate 3-D intra-cardiac echocardiography. Ultrasound Med. Biol..

[89] Shung K.K., Zippuro M. (1996). Ultrasonic transducers and arrays. IEEE Eng. Med. Biol. Mag..

[90] Chen C., Raghunathan S.B., Yu Z., Shabanimotlagh M., Chen Z., Chang Z.-y., Blaak S., Prins C., Ponte J., Noothout E. (2015). A prototype PZT matrix transducer with low-power integrated receive ASIC for 3-D transesophageal echocardiography. IEEE Trans. Ultrason. Ferroelectr. Freq. Control.

[91] Kang E., Ding Q., Shabanimotlagh M., Kruizinga P., Chang Z.-Y., Noothout E., Vos H.J., Bosch J.G., Verweij M.D., de Jong N. (2018). A Reconfigurable Ultrasound Transceiver ASIC With $24\times40 $ Elements for 3-D Carotid Artery Imaging. IEEE J. Solid-State Circuits.

[92] Chen C., Chen Z., Bera D., Raghunathan S.B., Shabanimotlagh M., Noothout E., Chang Z.-Y., Ponte J., Prins C., Vos H.J. (2017). A front-end ASIC with receive sub-array beamforming integrated with a 32 × 32 PZT matrix transducer for 3-D transesophageal echocardiography. IEEE J. Solid-State Circuits.

[93] McKeighen R. (1998). Design guidelines for medical ultrasonic arrays. Medical Imaging 1998: Ultrasonic Transducer Engineering.

[94] Chen R., Cabrera-Munoz N.E., Lam K.H., Hsu H.S., Zheng F., Zhou Q., Shung K.K. (2014). PMN-PT single-crystal high-frequency kerfless phased array. IEEE Trans. Ultrason. Ferroelectr. Freq. Control.

[95] Cannata J., Williams J., Shung K. (2005). A kerfless 30 MHz linear ultrasonic array. Proceedings of the IEEE Ultrasonics Symposium, 2005.

[96] Démoré C.E., Brown J.A., Lockwood G.R. (2006). Investigation of cross talk in kerfless annular arrays for high-frequency imaging. IEEE Trans. Ultrason. Ferroelectr. Freq. Control.

[97] Huff M. (2021). Recent advances in reactive ion etching and applications of high-aspect-ratio microfabrication. Micromachines.

[98] Peng J., Ma L., Li X., Tang H., Li Y., Chen S. (2018). A novel synchronous micro motor for intravascular ultrasound imaging. IEEE Trans. Biomed. Eng..

[99] Newnham R.E., Skinner D.P., Cross L.E. (1978). Connectivity and piezoelectric-pyroelectric composites. Mater. Res. Bull..

[100] Smith W.A., Auld B.A. (1991). Modeling 1-3 composite piezoelectrics: Thickness-mode oscillations. IEEE Trans. Ultrason. Ferroelectr. Freq. Control.

[101] Boujenoui A., El Atlas N., Bybi A., Reskal H., Elmaimouni L. (2025). Advances in Crosstalk Reduction Techniques for Ultrasonic Transducer Arrays. Sensors.

[102] Liu C., Djuth F.T., Zhou Q., Shung K.K. (2013). Micromachining techniques in developing high-frequency piezoelectric composite ultrasonic array transducers. IEEE Trans. Ultrason. Ferroelectr. Freq. Control.

[103] Li S. (2017). Micromachined Piezoelectric Material and Dual-Layer Transducers for Ultrasound Imaging.

[104] Bartel T., Müller S., Biviano A., Hahn R.T. (2014). Why is intracardiac echocardiography helpful? Benefits, costs, and how to learn. Eur. Heart J..

[105] Baruś P., Modrzewski J., Gumiężna K., Dunaj P., Głód M., Bednarek A., Wańha W., Roleder T., Kochman J., Tomaniak M. (2022). Comparative Appraisal of Intravascular Ultrasound and Optical Coherence Tomography in Invasive Coronary Imaging: 2022 Update. J. Clin. Med..

[106] Park D.Y., An S., Jolly N., Attanasio S., Yadav N., Gutierrez J.A., Nanna M.G., Rao S.V., Vij A. (2023). Comparison of intravascular ultrasound, optical coherence tomography, and conventional angiography-guided percutaneous coronary interventions: A systematic review, network meta-analysis, and meta-regression. Catheter. Cardiovasc. Interv..

[107] Huang X. (2015). CMOS Multimodal Sensor Based Lab-on-a-Chip System for Personalized Bio-Imaging Diagnosis. Ph.D. Thesis.

[108] He Y., Wan H., Jiang X., Peng C. (2023). Piezoelectric Micromachined Ultrasound Transducer Technology: Recent Advances and Applications. Biosensors.

[109] Abaravičius B. (2023). Development of Electronics for Microultrasound Capsule Endoscopy. Ph.D. Thesis.

[110] Hahn R.T., Mahmood F., Kodali S., Lang R., Monaghan M., Gillam L.D., Swaminathan M., Bonow R.O., Bardeleben R.S., Bax J.J. (2019). Core Competencies in Echocardiography for Imaging Structural Heart Disease Interventions: An Expert Consensus Statement. JACC Cardiovasc. Imaging.

[111] Kodeboina M., Piayda K., Jenniskens I., Vyas P., Chen S., Pesigan R.J., Ferko N., Patel B.P., Dobrin A., Habib J. (2023). Challenges and Burdens in the Coronary Artery Disease Care Pathway for Patients Undergoing Percutaneous Coronary Intervention: A Contemporary Narrative Review. Int. J. Environ. Res. Public Health.

[112] Wiegers S.E., Ryan T., Arrighi J.A., Brown S.M., Canaday B., Damp J.B., Diaz-Gomez J.L., Figueredo V.M., Garcia M.J., Gillam L.D. (2019). 2019 ACC/AHA/ASE Advanced Training Statement on Echocardiography (Revision of the 2003 ACC/AHA Clinical Competence Statement on Echocardiography): A Report of the ACC Competency Management Committee. Circ. Cardiovasc. Imaging.

[113] Alberti A., Giudice P., Gelera A., Stefanini L., Priest V., Simmonds M., Lee C., Wasserman M. (2016). Understanding the economic impact of intravascular ultrasound (IVUS). Eur. J. Health Econ..

[114] McLoughlin D. (2025). Development of an Automated Catheter Production Line with SCADA Integration for the Medical Device Industry. Bachelor’s Thesis.

[115] Wen Z.Y. (2025). Research on the Financial Impact of Centralized Procurement of Medical Consumables on Public Hospitals. Open J. Soc. Sci..

[116] Petrescu A., D’HOoge J., Voigt J. (2021). Concepts and applications of ultrafast cardiac ultrasound imaging. Echocardiography.

[117] Rathod K.S., Hamshere S.M., A Jones D., Mathur A. (2015). Intravascular Ultrasound Versus Optical Coherence Tomography for Coronary Artery Imaging—Apples and Oranges?. Interv. Cardiol..

[118] Xie Y., Han W., Wang S., Jia W., Wang Y., Li J., Chen B. (2025). Advantages of hybrid intravascular ultrasound-optical coherence tomography system in clinical practice. Front. Cardiovasc. Med..

[119] Shen H., Feng X.X., Guo Q.Y., Zhou Y.J. (2024). Updates of developments in interventional therapy for elderly patients with cardiovascular diseases. J. Geriatr. Cardiol..

[120] Tsigkas G., Nastouli K.M., Apostolos A., Spyropoulou P., Bozika M., Papafaklis M., Rouzi S., Tsimara E., Karanasos A., Mplani V. (2025). When Functional Assessment Meets Intravascular Imaging in Patients with Coronary Artery Disease. J. Cardiovasc. Dev. Dis..

[121] Wang J., Zheng Z., Chan J., Yeow J.T.W. (2020). Capacitive micromachined ultrasound transducers for intravascular ultrasound imaging. Microsyst. Nanoeng..

